# Coenzyme B_12_ ‐dependent and independent photoregulation of carotenogenesis across Myxococcales

**DOI:** 10.1111/1462-2920.15895

**Published:** 2022-01-27

**Authors:** Ricardo Pérez‐Castaño, Eva Bastida‐Martínez, Jesús Fernández‐Zapata, María del Carmen Polanco, María Luisa Galbis‐Martínez, Antonio A. Iniesta, Marta Fontes, S. Padmanabhan, Montserrat Elías‐Arnanz

**Affiliations:** ^1^ Departamento de Genética y Microbiología, Área de Genética (Unidad Asociada al IQFR‐CSIC), Facultad de Biología Universidad de Murcia Murcia 30100 Spain; ^2^ Instituto de Química Física “Rocasolano” Consejo Superior de Investigaciones Científicas Madrid 28006 Spain

## Abstract

Light‐induced carotenogenesis in *Myxococcus xanthus* is controlled by the B_12_‐based CarH repressor and photoreceptor, and by a separate intricate pathway involving singlet oxygen, the B_12_‐independent CarH paralogue CarA and various other proteins, some eukaryotic‐like. Whether other myxobacteria conserve these pathways and undergo photoregulated carotenogenesis is unknown. Here, comparative analyses across 27 Myxococcales genomes identified carotenogenic genes, albeit arranged differently, with *carH* often in their genomic vicinity, in all three Myxococcales suborders. However, CarA and its associated factors were found exclusively in suborder Cystobacterineae, with *carA*‐*carH* invariably in tandem in a syntenic carotenogenic operon, except for *Cystobacter/Melittangium*, which lack CarA but retain all other factors. We experimentally show B_12_‐mediated photoregulated carotenogenesis in representative myxobacteria, and a remarkably plastic CarH operator design and DNA binding across Myxococcales. Unlike the two characterized CarH from other phyla, which are tetrameric, *Cystobacter* CarH (the first myxobacterial homologue amenable to analysis *in vitro*) is a dimer that combines direct CarH‐like B_12_‐based photoregulation with CarA‐like DNA binding and inhibition by an antirepressor. This study provides new molecular insights into B_12_‐dependent photoreceptors. It further establishes the B_12_‐dependent pathway for photoregulated carotenogenesis as broadly prevalent across myxobacteria and its evolution, exclusively in one suborder, into a parallel complex B_12_‐independent circuit.

## Introduction

Light directs many key biological processes but can also cause photooxidative stress by generating highly reactive oxygen species (ROS), like singlet oxygen (^1^O_2_), which attack proteins, DNA and lipids (Ziegelhoffer and Donohue, 2009; Glaeser *et al*., 2011). Many organisms, including bacteria, combat photooxidative stress by inducing the synthesis of carotenoids, pigments that quench ROS (Armstrong, 1997; Ziegelhoffer and Donohue, 2009; Rodriguez‐Concepción *et al*., 2018; Sandmann, 2019). How light is sensed and signalled to trigger carotenogenesis has been studied in depth in *Myxococcus xanthus* (Elías‐Arnanz *et al*., 2011; Padmanabhan *et al*., 2021), a Gram‐negative soil bacterium of the order Myxococcales, currently included within the class Deltaproteobacteria but it was recently proposed that this order be moved to a new and independent phylum with the name Myxococcata (Waite *et al*., 2020). Members of this order, the myxobacteria, form an important group of ubiquitous, predominantly aerobic, soil bacteria that share complex lifestyles and several traits typical of eukaryotes, such as multicellular development, biosynthesis of specialized lipids and steroids, social behaviour, kin recognition (the ability of an individual cell or organism to identify others as self‐like), predation and motility (Muñoz‐Dorado *et al*., 2016; Cao and Wall, 2019; Gallego‐García *et al*., 2019; Hoshino and Gaucher, 2021). Given these eukaryotic‐like traits in myxobacteria, the Syntrophy hypothesis envisages an ancient myxobacterium at the origin of eukaryotes (López‐García and Moreira, 2020; Hoshino and Gaucher, 2021), and some phylogenetic signals supporting the theory can be traced to factors identified in *M. xanthus* light‐induced carotenogenesis. Hence, exploring how this response and its unique factors are conserved can provide valuable molecular as well as evolutionary insights, and here we examined this across Myxococcales.

**Fig. 1:**
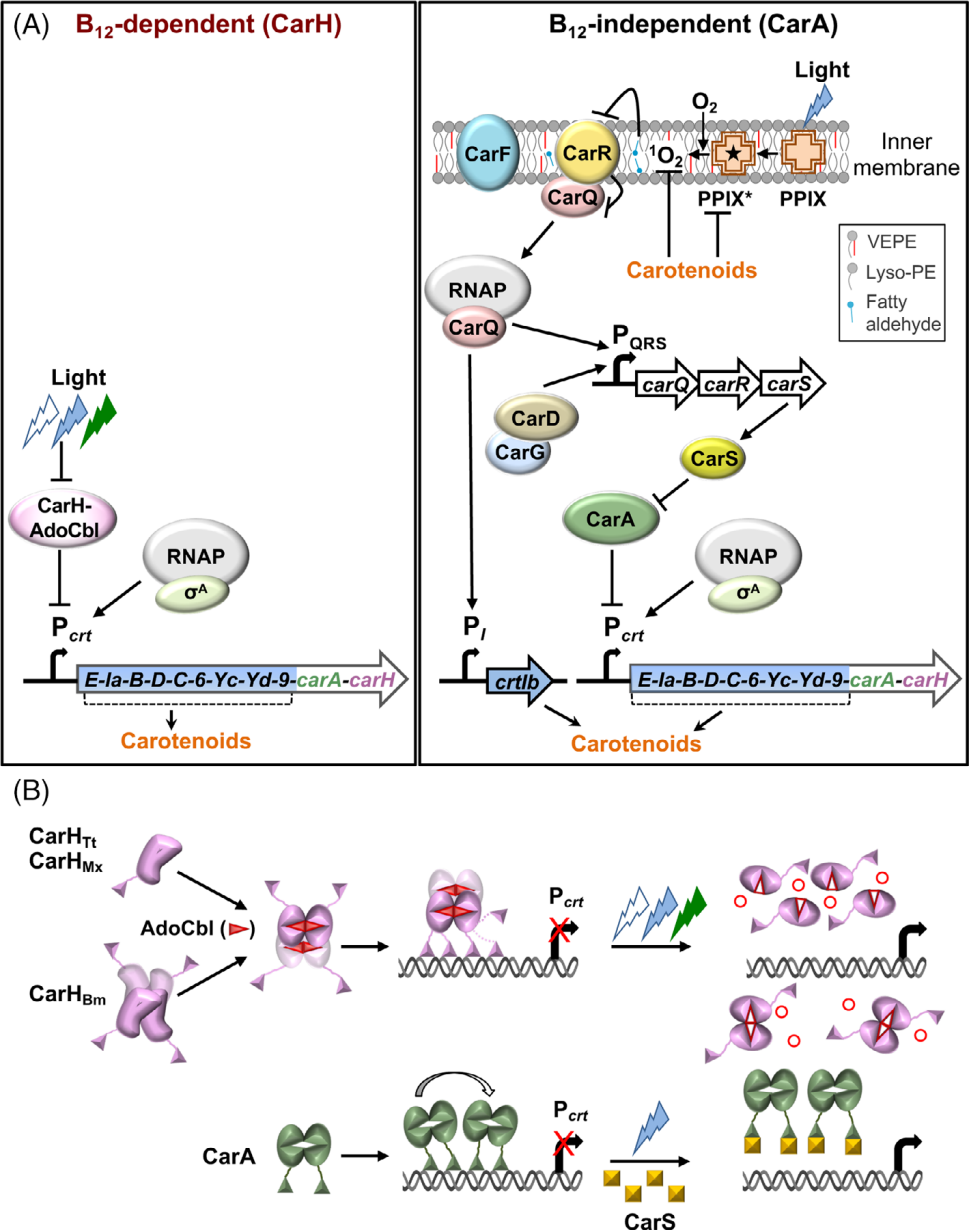
The two pathways for regulating *M. xanthus* light‐induced carotenogenesis. A. Left panel: B_12_‐dependent pathway. In the dark, CarH binds to its operator at P_*crt*_ (recognized by RNAP‐σ^A^) to repress expression of the carotenoid synthesis genes (*crtE–orf9*) of the *carB* operon. Light (UV/blue/green) disrupts CarH‐operator binding to allow transcription from P_*crt*_. Right panel: B_12_‐independent pathway. CarA represses P_*crt*_ in the dark and its action is counteracted by CarS, expressed when protoporphyrin IX (PPIX) exposed to blue light generates photoexcited PPIX (PPIX*) and ^1^O_2_. Plasmalogens (VEPE), whose biosynthesis requires CarF, are essential for inactivation of CarR by a mechanism that may involve VEPE cleavage by ^1^O_2_ to its lyso‐PE form and a fatty aldehyde (Gallego‐García *et al*., 2019) to free CarQ, which associates with RNAP to activate P_*I*_ and, together with the CarD–CarG complex, P_*QRS*_. B. Molecular mechanism of action of CarH and CarA. Top: Binding of AdoCbl to apoCarH (monomer or molten globule tetramer) forms active CarH repressor oligomers (tetramers: CarH_Tt_, CarH_Bm_; undefined oligomer: CarH_Mx_). Photolysis of the AdoCbl Co‐C bond liberates the Ado group as 4′, 5′‐anhydroadenosine (unfilled red circles) (Jost *et al*., 2015a) and disrupts CarH oligomers to monomers (CarH_Tt_, CarH_Mx_) or dimers (CarH_Bm_) with bound photolyzed AdoCbl (unfilled red triangles). Bottom: CarA dimers bind cooperatively to its operator, which overlaps with promoter elements at P_*crt*_, to repress expression in the dark. Blue light induces expression of CarS, which sequesters the CarA DNA binding domain leading to antirepression.

Two light sensing and signalling mechanisms control carotenogenesis in *M. xanthus* (Fig. 1A). One directly senses UV, blue or green light using a single protein, CarH_Mx_ (Mx for *M. xanthus*), which is the defining member of a large photoreceptor family whose chromophore is coenzyme B_12_ or 5′‐deoxyadenosylcobalamin (AdoCbl), a biological form of vitamin B_12_ (Fig. S1, Supporting Information) (Ortiz‐Guerrero *et al*., 2011; Jost *et al*., 2015b; Padmanabhan *et al*., 2017; Padmanabhan *et al*., 2019). The other more complex mechanism involves several factors including CarA_Mx_, an AdoCbl‐independent CarH_Mx_ paralogue and the extensive genetic rewiring that usually shapes the evolution of new regulatory networks (Perez and Groisman, 2009; Romero *et al*., 2012). It relies on ^1^O_2_ generated by blue light excitation of protoporphyrin IX, a photosensitizer and heme precursor (Fig. 1A) (Burchard and Dworkin, 1966; Galbis‐Martínez *et al*., 2012). The protein known to act earliest in this pathway is CarF (Fontes *et al*., 2003), which we recently unmasked as the long‐sought desaturase, widespread in metazoa but very rare in bacteria and required for the biosynthesis of plasmalogens, a special type of glycerophospholipids (Gallego‐García *et al*., 2019). These lipids and ^1^O_2_, through a still unknown mechanism, provoke the release of the extracytoplasmic function σ (ECF‐σ) factor CarQ from its membrane‐bound anti‐σ CarR (Galbis‐Martínez *et al*., 2012; Gallego‐García *et al*., 2019). CarQ then associates with RNA polymerase (RNAP) to turn on transcription of the *carQRS* operon (encoding CarQ, CarR and CarS, an antirepressor of CarA_Mx_) and of the carotenogenic gene *crtIb* (Martínez‐Argudo *et al*., 1998; Browning *et al*., 2003). This requires the CarD–CarG global regulatory complex, with some features reminiscent of multiprotein eukaryotic transcription complexes called enhanceosomes (Peñalver‐Mellado *et al*., 2006; García‐Heras *et al*., 2009; Elías‐Arnanz *et al*., 2010).

Both light signalling pathways converge at P_*crt*_ (or P_B_) (Fig. 1A), a promoter recognized by the primary σ^A^‐RNAP holoenzyme that drives expression of the *carB* operon, which encodes all the carotenoid synthesis enzymes except CrtIb, as well as CarH_Mx_ and CarA_Mx_ (López‐Rubio *et al*., 2004; Pérez‐Marín *et al*., 2008; Ortiz‐Guerrero *et al*., 2011). CarH_Mx_ requires AdoCbl for oligomerization (its precise oligomeric state is unknown) and DNA binding to repress transcription from P_*crt*_, and to be directly inactivated by light through the disassembly of the oligomer to monomers (Fig. 1B) (Pérez‐Marín *et al*., 2008; Ortiz‐Guerrero *et al*., 2011). In contrast to CarH_Mx_, homologues from species in other bacterial phyla, *Thermus thermophilus* (CarH_Tt_) and *Bacillus megaterium* (CarH_Bm_), have been purified and characterized *in vitro*, and crystal structures of the dark (free or DNA‐bound) and light‐exposed forms have been resolved for CarH_Tt_ (Ortiz‐Guerrero *et al*., 2011; Díez *et al*., 2013; Kutta *et al*., 2015; Jost *et al*., 2015a; Jost *et al*., 2015b; Fernández‐Zapata *et al*., 2018; Miller *et al*., 2020). In the dark, both proteins form AdoCbl‐bound tetramers that bind to operators comprising tandem 11‐bp direct repeats, three for CarH_Tt_ or four for CarH_Bm_. However, while apo and light‐exposed holoCarH_Tt_ are both monomers, like CarH_Mx_, apoCarH_Bm_ is a molten globule tetramer and light‐exposed holoCarH_Bm_ is a dimer (Fig. 1B). By contrast, CarA_Mx_ is a dimer in the dark or light, with or without B_12_, binds cooperatively to operator DNA as two dimers, and requires expression of CarS in the light for derepression (Fig. 1B) (López‐Rubio *et al*., 2004; Navarro‐Avilés *et al*., 2007; León *et al*., 2010; Ortiz‐Guerrero *et al*., 2011). CarH_Mx_ and CarA_Mx_ thus differ in AdoCbl‐dependence, oligomerization, and DNA binding modes to control the same promoter and target genes.

CarH homologues are widely distributed in several bacterial phyla, usually as a single copy (Padmanabhan *et al*., 2019). Hence, it is intriguing that CarH_Mx_ coexists with CarA_Mx_, whose function has required coevolution of a cohort of other unique factors. Such duplications and divergence, and their retention or loss are of great interest in understanding transcription factor evolution (Hittinger and Carroll, 2007; Chapal *et al*., 2019; Kuzmin *et al*., 2020). We therefore examined conservation of carotenogenesis and of the two *M. xanthus* photosensory regulatory pathways in all three Myxococcales suborders: Cystobacterineae (includes *M. xanthus*), Sorangiineae and Nannocystineae. This established that: (i) genes for carotenoid biosynthesis enzymes and for CarH are present in all myxobacterial genomes examined except in *Anaeromyxobacter dehalogenans* and *Vulgatibacter incomptus*, both with reduced genomes; (ii) that *carH* in Cystobacterineae always occurs in a large syntenic cluster of carotenogenic genes that in some species also contains, interestingly, a gene for 3‐hydroxy‐3‐methyl‐glutaryl‐CoA reductase or HMGR (a separate syntenic operon encodes the primary HMGR and other enzymes of the mevalonate or MVA pathway for the synthesis of isoprenoids, the precursors of carotenoids); (iii) even though carotenogenic genes are rearranged in Sorangiineae and Nannocystineae, *carH* frequently occurs in the genomic vicinity. We provide evidence supporting B_12_‐dependent photoregulation of carotenogenesis in representative myxobacteria from all three suborders and its likely mediation by CarH, whose operator design and DNA binding are, we show, remarkably plastic. The parallel CarA pathway and all its associated players appear strictly confined to suborder Cystobacterineae, among which the *Cystobacter/Melittangium* genera have CarH and all factors of the CarA pathway yet, surprisingly, not CarA. We purified and characterized *Cystobacter* CarH (the one myxobacterial homologue thus far amenable to analysis *in vitro*) and show that it differs from the tetrameric CarH_Tt_ and CarH_Bm_, the only others that have been characterized *in vitro* and which belong to different phyla. Thus, *Cystobacter* CarH acts as a dimer with CarH‐like B_12_‐dependent photosensory mode of action as well as CarA‐like DNA binding and its antirepression by CarS. Altogether, this work reveals that B_12_‐based photoregulation of carotenogenesis pervades across Myxococcales and provides molecular‐evolutionary insights into this photoprotective response in these bacteria.

## Results

### CarH is present in all three myxobacterial suborders and CarA only in Cystobacterineae

**Fig. 2:**
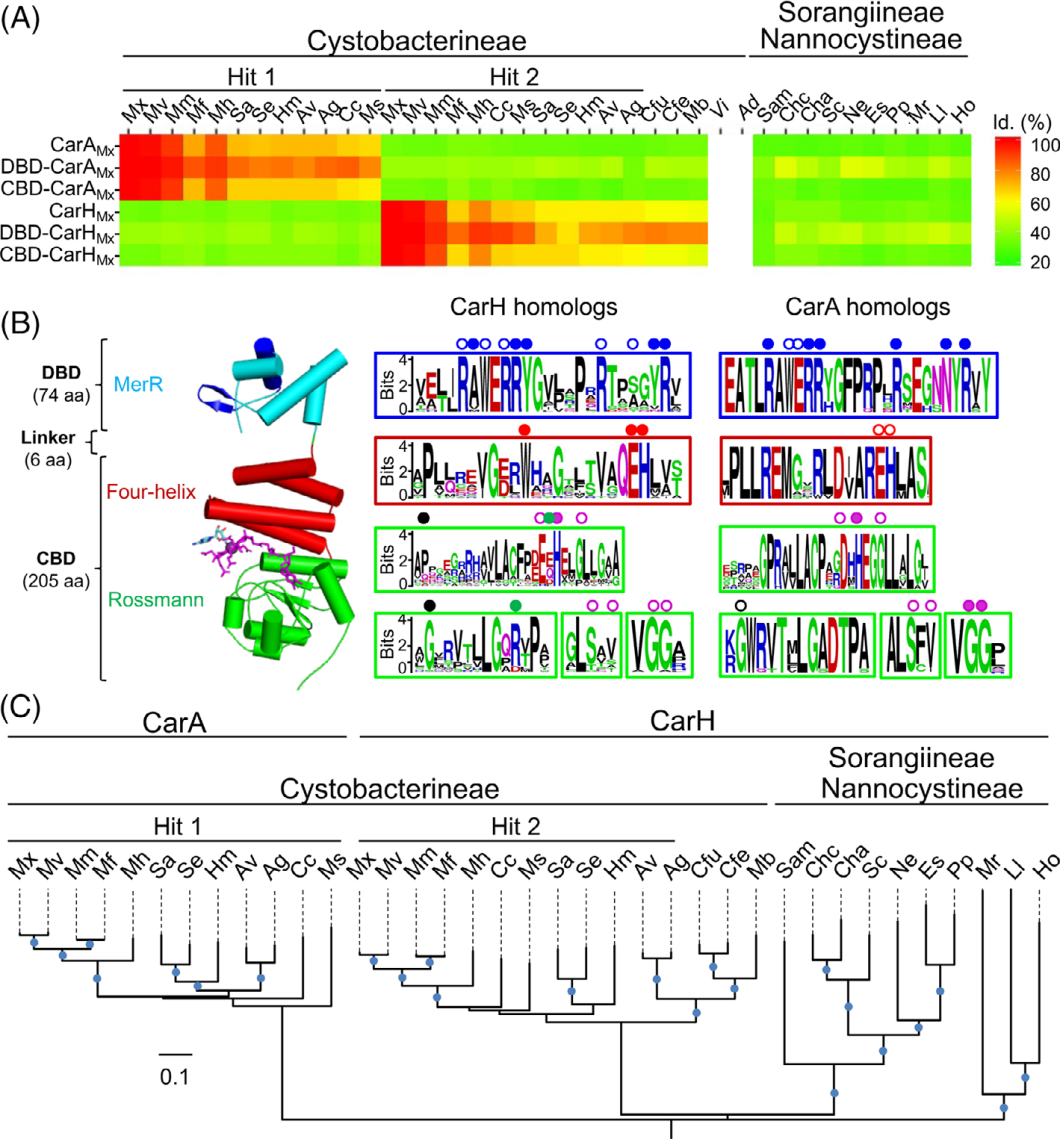
CarA and CarH homologues in Myxococcales. A. Heat map of the sequence identities of myxobacterial CarH/A homologues relative to CarA_Mx_, CarH_Mx_ and their DBDs or CBDs (see Table S1 for species abbreviations). B. Left: Structure of the CarH_Tt_ protomer (PDB code: 5C8D) with its DBD (DNA recognition helix and wing in dark blue) and its CBD, with bound AdoCbl (sticks in magenta with upper axial Ado group in cyan). Right: Weblogos of myxobacterial CarH/A homologues alongside the corresponding subdomains in CarH_Tt_. Dots indicate DNA contacts (blue), Wx_9_EH motif (red), lower axial B_12_‐binding motif (magenta), dimer contacts (green) and dimer‐dimer interface (black), based on the CarH_Tt_ and DBD‐CarA_Mx_ structures, with filled dots indicating residues tested by mutational analysis (Navarro‐Avilés *et al*., 2007; Jost *et al*., 2015b). C. Maximum likelihood phylogeny (blue dots >80% bootstrap values) for myxobacterial CarH and CarA homologues.

Myxococcales currently comprise three suborders and 10 families, and we examined 27 complete, publicly accessible myxobacterial genomes for genes encoding CarA/CarH homologues and other elements of photoinduced carotenogenesis identified in *M. xanthus* (Table S1). These myxobacteria are soil‐dwelling, except for the aquatic *Enhygromyxa salina*, *Haliangium ochraceum* and *Plesiocystis pacifica,* and obligate aerobes except for *A. dehalogenans*, a facultative anaerobe. Their GC‐rich (66%–75%) genomes are among the largest in bacteria (~9–16 Mb) but are reduced, presumably by extensive gene loss, in *V. incomptus* (~4.4 Mb) and *A. dehalogenans* (~5 Mb) (Huntley *et al*., 2011; Yamamoto *et al*., 2014). BLAST searches of these genomes using CarA_Mx_ or CarH_Mx_ (35% identical) as query yielded one hit in Sorangiineae and Nannocystineae, and two hits in Cystobacterineae except for a single hit in *Cystobacter ferrugineus*, *Cystobacter fuscus* and *Melittangium boletus*, and none in *A. dehalogenans* or *V. incomptus*. In Cystobacterineae with two hits, one was most similar to CarA_Mx_ (70%–98% identity, versus 33%–38% to CarH_Mx_) and the other to CarH_Mx_ (62%–97% identity, versus 32%–34% to CarA_Mx_), suggesting the first and second sets to be CarA_Mx_ and CarH_Mx_ orthologs respectively (Fig. 2A and Table S2). Interestingly, the single hits in *C. ferrugineus*, *C. fuscus* and *M. boletus* were most similar to CarH_Mx_ (57%–60% identity) than to CarA_Mx_ (33%–36%), while sequence identities to CarH_Mx_ (32%–39%) or to CarA_Mx_ (29%–34%) were comparable for hits in Sorangiineae and Nannocystineae (Fig. 2A and Table S2).

Whether the observed hits are *bona fide* CarA_Mx_ or CarH_Mx_ orthologs was assessed further by sequence analysis and experimentally. CarA_Mx_ and CarH_Mx_ have similar architectures, with an N‐terminal MerR‐type winged‐helix DNA binding domain (DBD) and a C‐terminal cobalamin‐binding domain (CBD) (Fig. 2B). Sequence comparisons revealed patterns in each of these domains closely parallel to those for whole CarA_Mx_ or CarH_Mx_, with the highest sequence identities between CarA_Mx_ domains and likely CarA homologues, and CarH_Mx_ domains and likely CarH homologues within Cystobacterineae (Fig. 2A). Our previous studies of CarH_Tt_, CarH_Bm_, CarA_Mx_ and CarH_Mx_ established key motifs in both the DBD and CBD (Navarro‐Avilés *et al*., 2007; Jost *et al*., 2015b; Fernández‐Zapata *et al*., 2018). In the CarH_Tt_ structure, the CBD comprises: (i) an N‐terminal four‐helix bundle subdomain with a Wx_9_EH motif (x = any residue), conserved in CarH_Mx_ and CarH_Bm_, which contacts the upper axial Ado group of CarH‐bound AdoCbl and is crucial for CarH photoreceptor activity; (ii) a C‐terminal five‐stranded α/β Rossmann module housing the classic D/ExHx_2_G/Px_41_SxT/Vx_22–27_GG B_12_‐binding motif, whose His supplies the lower axial ligand of bound AdoCbl (Fig. S1B and Fig. S2) (Jost *et al*., 2015b; Fernández‐Zapata *et al*., 2018). The CarA_Mx_ CBD retains the B_12_‐binding motif and EH of the Wx_9_EH motif but lacks the Trp and four flanking residues (Fig. S2). Accordingly, whether an intact Wx_9_EH motif co‐occurs or not with the classic B_12_‐binding motif may help distinguish CarH and CarA homologues. Classification of the Cystobacterineae homologues applying this criterion matched that based on overall sequence homology to CarA_Mx_ and CarH_Mx_; moreover, all the single Sorangiineae and Nannocystineae homologues could be assigned as CarH. Consistent with these assignments, CarA homologues form a monophyletic clade within Cystobacterineae, separate from the coexisting CarH or the single Sorangiineae or Nannocystineae homologues (Fig. 2C). Sequence alignment of myxobacterial homologues thus classified as CarA or CarH yielded logos for the signature motifs in each domain shown in Fig. 2B. Besides the presence of a classic B_12_‐binding motif in all myxobacterial homologues, and an intact Wx_9_EH motif only in CarH homologues, the logos revealed that the RxWERRY motif in the DNA recognition α‐helix and several other residues implicated in DNA binding are conserved in the predicted DBDs of both CarH and CarA homologues, suggesting a common mode of DNA recognition. Also, many CarH homologues have: Trp of the Wx_9_EH motif followed by a His, which in light‐exposed CarH_Tt_ is the upper ligand in a bis‐His‐cobalt linkage; a Glu‐Arg or Asp‐Arg pair equivalent to the crucial Arg‐Asp salt‐bridge pair of the CarH_Tt_ dimer interface; and one of the two Gly at the dimer–dimer interface important in CarH_Tt_ tetramer assembly (the other Gly is often Pro) conserved in CarH as well as CarA homologues (Fig. 2B).

**Fig. 3:**
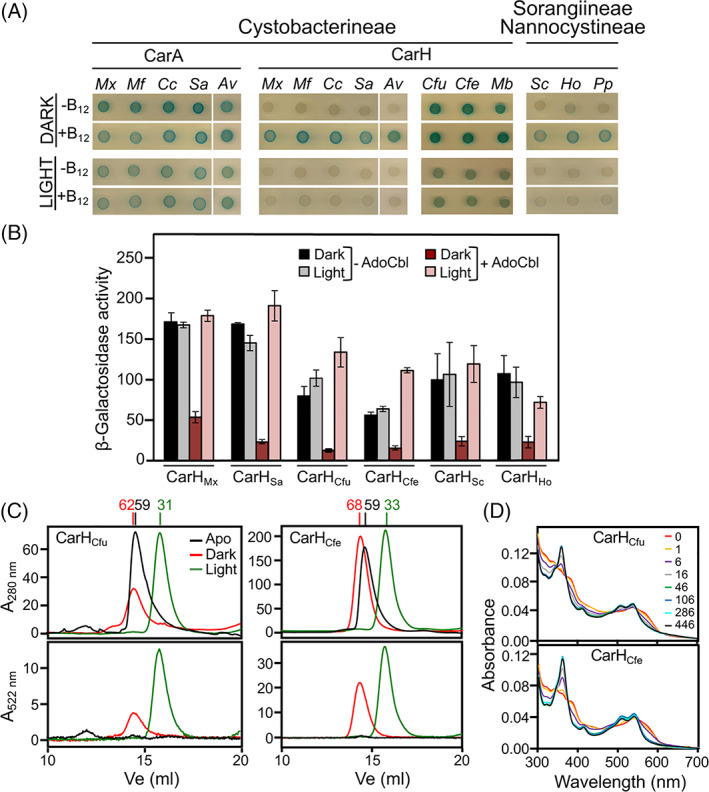
Experimental tests of myxobacterial CarH/CarA assignments. A. BACTH analysis of the effect of B_12_ and light on self‐interaction of various myxobacterial CarH/CarA homologues. Cells expressing T25 and T18 fusions of CarH or CarA were spotted on X‐Gal‐LB plates with or without vitamin B_12_ and incubated for 48 h in the dark or exposed to light. B. Complementation analysis in *M. xanthus*. Reporter P_*crt* Mx_::*lacZ* expression (specific β‐galactosidase activity shown as the mean and standard error of three independent measurements) in the dark and in the light with AdoCbl present or absent in *M. xanthus* strain MR2649 bearing the indicated myxobacterial homologue, expressed from a vanillate‐inducible promoter using vanillate at 100 μM (CarH_Mx_, CarH_Sa_, CarH_Cfu_, CarH_Cfe_), 2 μM (CarH_Sc_) or 500 μM (CarH_Ho_). C. Size‐exclusion chromatography elution profiles tracked using absorbance at 280 or 522 nm for CarH_Cfu_ and CarH_Cfe_ in the apo form in the dark, and with AdoCbl present in the dark (red) or after 5‐min exposure to green light (green). Apparent *M*_r_ (kDa) for each eluted peak is on top. D. UV–visible absorbance spectra for the photoconversion of AdoCbl‐bound CarH_Cfu_ and CarH_Cfe_ upon stepwise illumination with green light for the times (in seconds) indicated.

To experimentally test our assignments, we first analysed several CarA and CarH homologues for self‐interactions using the bacterial adenylate cyclase two‐hybrid (BACTH) assay in *E. coli* (which cannot synthesize B_12_ *de novo* but can take up and convert exogenous B_12_ to AdoCbl for use *in vivo*). This assay has demonstrated that CarH_Mx_ self‐interacts via its CBD only in the dark and when B_12_ is present, whereas CarA_Mx_ self‐interacts in the dark or light, irrespective of the presence or not of B_12_ (Ortiz‐Guerrero *et al*., 2011). In the BACTH assay, the homologues we assigned as CarA and CarH behaved like CarA_Mx_ and CarH_Mx_, respectively, except for the sole *C. ferrugineus*, *C. fuscus* and *M. boletus* homologues, whose behaviour mirrored CarA_Mx_ rather than CarH_Mx_ (Fig. 3A). Negative controls with only one fusion protein ruled out autoactivation in the behaviour of the *C. fuscus*, *C. ferrugineus* and *M. boletus* homologues (Fig. S3).

We next checked our assignments by testing if CarH_Mx_ could be functionally replaced by representative CarH homologues: CarH_Sa_, CarH_Cfu_ and CarH_Cfe_ from *S. aurantiaca*, *C. fuscus* and *C. ferrugineus*, respectively, outside the *Myxococcus–Corallococcus* branch in Cystobacterineae; CarH_Sc_ from *S. cellulosum* in Sorangiineae; and CarH_Ho_ from *H. ochraceum* in Nannocystineae. Each homologue was expressed from a vanillate‐inducible promoter in an *M. xanthus* strain (MR2649) with a P_*crt* Mx_::*lacZ* transcriptional fusion and which lacks endogenous CarH, CarA, CarS and PduO, the ATP:corrinoid adenosyltransferase essential for AdoCbl generation, to enable controlled exogenous AdoCbl supply. All myxobacterial homologues tested, including CarH_Cfu_ and CarH_Cfe_, downregulated reporter P_*crt* Mx_::*lacZ* expression in the dark when AdoCbl was present but not when absent or when the cells were exposed to light, and hence are functionally equivalent to CarH_Mx_ (Fig. 3B).

We could purify CarH_Cfu_ and CarH_Cfe_ in the apo form as soluble proteins, unlike other myxobacterial CarH tested, and analyse them *in vitro*. Consistent with the BACTH assay, in size exclusion chromatography apoCarH_Cfu_ and apoCarH_Cfe_ eluted predominantly as dimers (*M*_r_  ~59 kDa, compared to ~35 kDa determined from the sequence or by mass spectrometry) (Fig. 3C). In the presence of AdoCbl, both continued to elute as dimers in the dark (*M*_r_ ~62 and ~68 kDa respectively), but as monomers when exposed to light (*M*_r_ ~31 and ~33 kDa respectively) (Fig. 3C), in contrast to their self‐interaction in BACTH analysis. In the latter assay, photolysis of intracellular AdoCbl and consequent poor binding to the two homologues probably causes them to persist as apoproteins and self‐interact. The absorbance of the holoproteins at 522 nm (besides at 280 nm) indicated that both the dark and light forms bind to cobalamin (Fig. 3C) at ~1:1 stoichiometry. Moreover, the dark to light photoconversion of their AdoCbl‐bound form upon stepwise illumination with green light (Fig. 3D) was similar to CarH_Tt_ or CarH_Bm_ (Kutta *et al*., 2015; Fernández‐Zapata *et al*., 2018; Miller *et al*., 2020). CarH_Cfu_ and CarH_Cfe_ are therefore AdoCbl‐based CarH photoreceptors but transition from an apodimer to a holodimer to a photolyzed monomer, in a new twist to the oligomeric plasticity of these photoreceptors. Altogether, our analyses indicate that CarH occurs in all three myxobacterial suborders, but CarA only in Cystobacterineae and always with CarH.

### Genes for CarA, CarH and carotenoid synthesis usually occur in genomic proximity and occasionally linked to that for HMGR

**Fig. 4:**
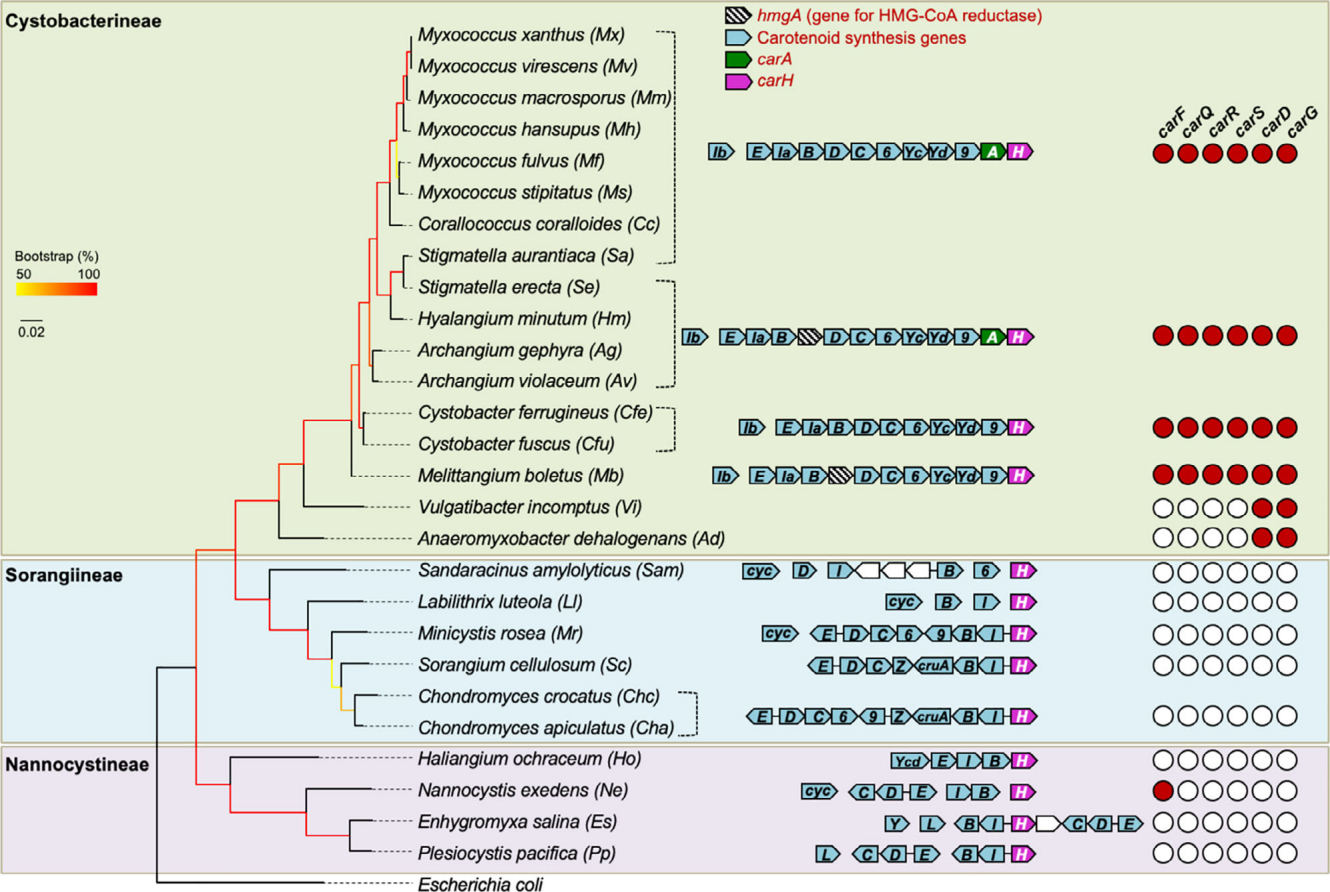
CarH, CarA and other factors in the B_12_‐independent pathway identified from myxobacterial genome analysis. The 16S rRNA‐based phylogenetic tree for myxobacteria highlighting the three suborders and species, and the genes for *carA* (*A*), *carH* (*H*), and carotenoid synthesis. *B*: *crtB* (phytoene synthase); *C*: *crtC* (neurosporene hydroxylase); *cruA*: CruA‐type lycopene cyclase; *cyc*: possible lycopene cyclase*; D*: *crtD* (hydroxyneurosporene dehydrogenase); *E*: *crtE* (geranyl geranyl pyrophosphate synthase); *I/Ia/Ib*: *crtI* (phytoene desaturase); *L*: *crtL* (CrtL‐type lycopene cyclase); *Y*: lycopene cyclase fused to CrtU; *Yc*: *crtYc* (CrtYc subunit of heterodimeric lycopene cyclase); *Yd*: *crtYd* (CrtYd subunit of heterodimeric lycopene cyclase); *Ycd*: *crtYcd* (heterodimeric fusion type CrtYcd lycopene cyclase); *Z*: *crtZ* (β‐carotene hydroxylase); *6*: *orf6* (possible glycosyltransferase); *9*: *orf9* (possible acyltransferase). Genes *carF*, *carQ*, *carR*, *carS*, *carD* and *carG* are indicated by the red circles if present and by unfilled circles if absent.

To determine whether the link between carotenogenesis and CarH/A is maintained in other myxobacteria, we analysed the genomic context of *carA* and *carH*, and performed a search for carotenoid synthesis genes. We found the latter in all myxobacteria examined except *A. dehalogenans* and *V. incomptus*, which also lack *carA* and *carH* (Fig. 4 and Table S2). Within Cystobacterineae, one phylogenetic branch that includes all *Myxococcus*, *Corallococcus*, *Stigmatella*, *Hyalangium* and *Archangium* species presents a genomic arrangement of carotenoid genes (with *crtIb* also at a different genomic location), *carA* and *carH* matching that in *M. xanthus*. Synteny is conserved in the branch containing *C. ferrugineus*, *C. fuscus* and *M. boletus*, except for the absence of *carA* (Fig. 4). In *M. xanthus*, the primary carotenoid end product in light‐grown cells is myxobacton ester (a monocyclic carotenoid), although significant amounts of phytoene (the first C_40_ compound in the biosynthetic pathway) are also found (Fig. S4) (Iniesta *et al*., 2007). This biosynthetic pathway is likely conserved in the other Cystobacterineae with the same set of carotenogenic genes as *M. xanthus*.

Interestingly, five species within Cystobacterineae (*Stigmatella erecta*, *Hyalangium minutum*, *Archangium gephyra*, *Archangium violaceum* and *M. boletus*) have a gene encoding HMGR between *crtB* and *crtD* in the syntenic carotenoid cluster (Fig. 4 and Table S2). HMGR is an enzyme that acts early in the MVA pathway for producing isopentenyl pyrophosphate (IPP) and its dimethylallyl pyrophosphate (DMAPP) isomer, the C_5_ building blocks for the biosynthesis of isoprenoids, including carotenoids (Fig. S4). The MVA pathway is one of the two known for isoprenoid biosynthesis and is used exclusively by archaea, fungi and animals; within bacteria, it is used only by a few species, since the majority employ the alternative methylerythritol 4‐phosphate (MEP) pathway (Moise *et al*., 2014; Rodriguez‐Concepción *et al*., 2018; Hoshino and Gaucher, 2021). Our observation that a gene for HMGR lies within the carotenogenic cluster in some myxobacteria prompted us to examine isoprenoid biosynthesis pathways in the species studied here. Interestingly, while *A. dehalogenans* and *V. incomptus* have the complete gene set for the MEP pathway, all the remaining myxobacteria have MVA pathway genes and are thus among the minority of bacteria employing this typically eukaryotic route for isoprenoid biosynthesis (Hoshino and Gaucher, 2021). The gene for the primary HMGR, a class II type conserved in all myxobacteria (except the two mentioned earlier) tends to occur in a syntenic cluster with genes for enzymes catalysing the subsequent four steps of the MVA pathway (Figs. S4 and S5A, and Table S2). The primary HMGRs in *S. erecta*, *H. minutum*, *A. gephyra*, *A. violaceum* and *M. boletus* share 46%–51% sequence identity (all catalytic residues are conserved; Fig. S5B) with the second HMGRs encoded by the gene within the carotenogenic cluster.

Compared to Cystobacterineae, carotenoid synthesis genes in Sorangiineae and Nannocystineae are often rearranged and/or redistributed as various clusters, with *crtI* existing as a single copy (Fig. 4). Even so, syntenic blocks of carotenogenic genes with *carH* are observed in the Sorangiineae examined (except for *Sandaracinus amylolyticus* and *Labilithrix luteola*), which are split into smaller blocks in Nannocystineae (Fig. 4). Some species appear to conserve only genes acting in the early biosynthetic steps, suggesting that their carotenoid end products may differ. Also, while *M. xanthus* and other Cystobacterineae (based on protein conservation relative to *M. xanthus*) use a heterodimeric CrtYc–CrtYd cyclase (Iniesta *et al*., 2008) to catalyse the formation of cyclic carotenoids from acyclic lycopene (Fig. S4), lycopene cyclases of the CruA family were found in Sorangiineae and of the CrtL family in Nannocystineae (Fig. 4). CruA‐type cyclases typically occur in green sulfur bacteria and cyanobacteria, and the CrtL‐type in cyanobacteria and plants (Maresca *et al*., 2007). Despite these differences, *carH* was generally found adjacent to carotenoid synthesis genes in Sorangiineae and Nannocystineae. Neither of these two suborders has a gene encoding HMGR within a carotenoid synthesis gene cluster. Their only gene for HMGR occurs with MVA pathway genes, and a second copy is found only in *H. ochraceum* and *S. amylolyticus* (Fig. S5A). Interestingly, in the latter and in Nannocystineae, the MVA cluster includes genes for the synthesis of hopanoids (polycyclic triterpenes, like sterols, with six IPP units), which have been functionally and evolutionarily linked to carotenoids (Santana‐Molina *et al*., 2020). All the myxobacterial genomes analysed, excluding the downsized ones in *A. dehalogenans* and *V. incomptus*, thus have carotenoid synthesis genes, with proximal *carH* and/or *carA* in the majority, hinting at light and CarH/CarA‐mediated regulation of carotenogenesis in these myxobacteria, as in *M. xanthus*.

### The B_12_ ‐independent CarA pathway evolved solely in Cystobacterineae

We next surveyed myxobacterial genomes for the other essential factors of the *M. xanthus* B_12_‐independent, CarA‐dependent regulatory pathway (CarF, CarQ, CarR, CarS, CarD and CarG), which have singular features. Our search for myxobacterial homologues of CarF_Mx_, the desaturase required for the biosynthesis of plasmalogen lipids typically found in metazoa (Gallego‐García *et al*., 2019), yielded single hits (each encoded by an isolated gene transcribed divergently relative to its flanking genes) in all Cystobacterineae except *A. dehalogenans* and *V. incomptus*, and in the other two suborders only in *N. exedens* (Fig. 4, Fig. S6 and Table S2). All these hits share sequence identities to CarF_Mx_ of ≥ 70% among Cystobacterineae (Fig. S6A) and 54% for that in *N. exedens*, and they conserve the nine histidines shown to be essential for function in CarF_Mx_ and its metazoan homologues (Gallego‐García *et al*., 2019).

Of the three proteins encoded by the *carQRS* operon, CarQ belongs to the large ECF‐σ factor family, whose members are widespread, because use of such alternative σ factors constitutes a fundamental mechanism of signal transduction across eubacteria. Moreover, the number of ECF‐σ factors in myxobacteria tends to be high (∼45 in *M. xanthus*, ∼87 in *S. cellulosum*, ∼118 in *P. pacifica*) (Abellón‐Ruiz *et al*., 2014). Consequently, unequivocal assignment of genuine CarQ orthologs relied on the simultaneous presence of the far more distinct CarR and CarS. CarR has the unusual six transmembrane‐helix topology of DUF1109 family anti‐σ factors largely restricted to Proteobacteria, some of which have been implicated in heavy metal or oxidative stress responses (Kohler *et al*., 2012; Masloboeva *et al*., 2012). However, CarR and its myxobacterial orthologs share low sequence similarity to other DUF1109 factors. Interestingly, CarR is a dual response anti‐σ that can be inactivated by the joint action of light‐induced ^1^O_2_ and plasmalogens (Galbis‐Martínez *et al*., 2012; Gallego‐García *et al*., 2019) as well as by copper (Moraleda‐Muñoz *et al*., 2005). On the other hand, the CarS antirepressor is a DNA mimic (León *et al*., 2010) with no sequence homologues beyond Myxococcales. We identified CarR and CarS homologues only in Cystobacterineae and, except for *A. dehalogenans* and *V. incomptus*, in all species analysed in this suborder, with *carR* and *carS* always with *carQ* as a *carQRS* operon, as in *M. xanthus* (Fig. 4, Fig. S6 and Table S2).

Our earlier studies established the unique two‐domain architecture of CarD, comprising an N‐terminal RNAP‐interacting domain, widely distributed among bacteria, which in CarD also interacts with CarG; a C‐terminal K/A/P‐rich DNA‐binding domain akin to eukaryotic high mobility group A (HMGA) proteins, with four or three repeats of their characteristic AT‐hook motifs in CarD_Mx_ and CarD_Sa_ respectively, but which lacks the AT‐hooks and resembles the histone H1 C‐terminal region in CarD_Ad_ (Padmanabhan *et al*., 2001; Cayuela *et al*., 2003; García‐Heras *et al*., 2009; García‐Moreno *et al*., 2010). On the other hand, an inactive version of the metzincin‐type zinc‐binding metalloprotease motif typically found in Archaea and vertebrates (Marino‐Puertas *et al*., 2017) occurs in CarG, which regulates gene expression by interacting with another transcription factor (CarD) rather than directly with DNA (Peñalver‐Mellado *et al*., 2006). Our search identified CarD and CarG orthologs (encoded in an operon, as in *M. xanthus*) only among Myxococcales and exclusively in Cystobacterineae, and in every species analysed in this suborder (Fig. 4, Fig. S6 and Table S2). Among CarD orthologs, only CarD_Vi_ has an H1‐like C‐terminal domain‐like CarD_Ad_, the rest having four AT‐hooks or three (in CarD_Sa_, CarD_Se_ and CarD_Hm_).

In sum, given the absence of all six factors among Sorangiineae and Nannocystineae (except for CarF in *N. exedens*), the entire B_12_‐independent pathway appears to have evolved uniquely in Cystobacterineae, in parallel with the advent of CarA. It is therefore intriguing that all six factors occur in *C. ferrugineus*, *C. fuscus* and *M. boletus*, even though none of the three species have CarA. Despite lacking CarH as well as CarA and four of its associated factors, *A. dehalogenans* and *V. incomptus* retain CarD and CarG, possibly because the two proteins act as a global regulatory complex in processes other than light‐induced carotenogenesis (García‐Heras *et al*., 2009; Abellón‐Ruiz *et al*., 2014; Bernal‐Bernal *et al*., 2018).

### P_*crt*_ operator design in Cystobacterineae

Most Cystobacterineae conserve the *M. xanthus* structural and regulatory genes for light‐induced carotenogenesis, as well as their genomic arrangement (Fig. 4). This suggests a common strategy to control carotenoid synthesis, with both CarA and CarH‐dependent pathways in the *Myxococcus*, *Corallococcus*, *Stigmatella*, *Hyalangium* and *Archangium* species, but only the latter in *C. ferrugineus*, *C. fuscus* and *M. boletus*. A common mode of regulation would likely be reflected by DNA sequence conservation around P_*crt*_, which we analysed next.

**Fig. 5:**
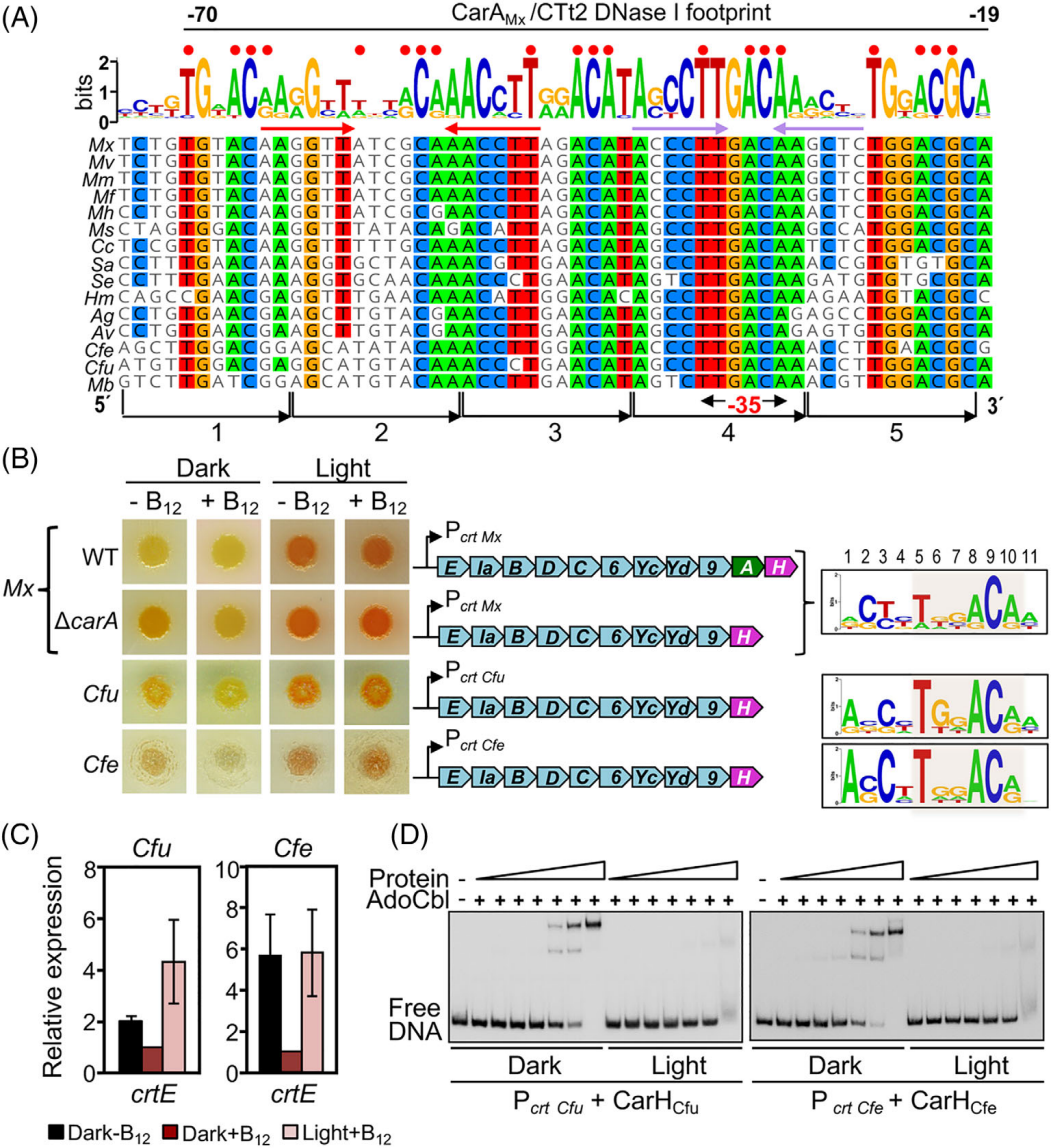
B_12_‐dependent light‐induced carotenogenesis and CarH DNA binding in *Cystobacter*. A. Alignment of the CarA_Mx_/CarH_Mx_ operator DNA sequence (sense strand) at P_*crt* Mx_ and the corresponding segments for Cystobacterineae species with CarH. Red dots align with T and ACA of the consensus DR motif, and the span of the CarA_Mx_/CTt2 DNase I footprint at P_*crt* Mx_ is indicated; below the logo is a perfect inverted repeat (red arrows) and a less perfect one (purple arrows) found in some *Myxococcus* species. The **−**35 promoter element within DR4 and the remaining four 11‐bp repeats are indicated at the bottom. B. Left: Colour of cell culture spots on plates with or without vitamin B_12_ incubated for 48 h in the dark or in the light for *M. xanthus* (wild‐type and Δ*carA* strains), *C. fuscus* and *C. ferrugineus* with the corresponding carotenogenic gene cluster and sequence logo (T*nn*ACA segment or equivalent shaded) of the five direct repeats at P_*crt*_. C. Expression of *crtE* from *C. fuscus* and *C. ferrugineus* measured by qRT‐PCR with *rpoD* as the reference gene reported relative to the level in the dark with vitamin B_12_ present (mean and standard error from three biological replicates). D. Representative EMSA for the binding of probes P_*crt* Cfu_ and P_*crt* Cfe_ to increasing concentrations of AdoCbl‐bound CarH_Cfu_ or CarH_Cfe_ (0.1, 0.25, 0.5, 1, 5, 10, 100 nM), respectively, in the dark or after 5‐min exposure to green light.

DNA binding assays with purified CarA_Mx_ revealed its binding as two dimers in a stepwise cooperative manner to an ~55 bp operator at P_*crt* Mx_, with the proposed CarA_Mx_ binding site containing two inverted repeats (Fig. 5A): a perfect one upstream of the **−**35 promoter region and a second imperfect one overlapping with it (López‐Rubio *et al*., 2004). However, among Cystobacterineae with CarA, only *Myxococcus* species and *C. coralloides* conserve a perfect or near‐perfect match to the *M. xanthus* inverted repeats, which steadily degenerate in the rest. CarH_Tt_ and CarH_Bm_, on the other hand, have been shown to bind as AdoCbl‐bound tetramers to tandem 11‐bp direct repeats (DRs) with the sequence 5´‐*nnnn*T*nn*ACA*n*‐3′ (*n* is any base). While CarH_Tt_ uses three of its DBDs to bind three tandem DRs, one of which includes the **−**35 promoter element, CarH_Bm_ binds to operators comprising four such tandem DRs, or three DRs and one pseudorepeat (hereafter dr, where the T*nn*ACA motif is not fully conserved), which overlap with the **−** 35 or **−** 10 promoter elements (Jost *et al*., 2015b; Fernández‐Zapata *et al*., 2018). Soluble native CarH_Mx_ has resisted purification for analysis *in vitro*, but a functionally equivalent chimeric protein (CTt2) with CarH_Mx_ CBD replaced by that of CarH_Tt_ recognized the same ~55‐bp operator at P_*crt* Mx_ as CarA_Mx_ (Ortiz‐Guerrero *et al*., 2011; Padmanabhan *et al*., 2017). Inspection of the ~55‐bp DNA segment at P_*crt* Mx_ revealed three 11‐bp DRs (DR1, DR3 and DR4) conforming to those identified for CarH_Tt_ and CarH_Bm_, and two drs (dr2 and dr5), the **−**35 TTGACA promoter element being within DR4 (Fig. 5A and Fig. S7). Sequence comparisons revealed a similar DR1‐dr2‐DR3‐DR4‐dr5 arrangement in all other *Myxococcus* species, *C. coralloides*, *S. aurantiaca* and *S. erecta*, which is dr1‐DR2‐DR3‐DR4‐dr5 in *H. minutum*, *C. fuscus*, *C. ferrugineus* and *M. boletus*, and dr1‐dr2‐DR3‐DR4‐dr5 in *A. gephyra* and *A. violaceum* (Fig. 5A and Fig. S7). DNA sequence logos from aligning the repeats at each promoter highlight the T*nn*ACA motif, but also reveal variations between closely related species, particularly in the less conserved bases that can contribute additional sequence information to fine–tune DNA binding affinity and specificity (Fig. 5A and Fig. S7) (Jost *et al*., 2015b; Fernández‐Zapata *et al*., 2018). Yet, despite these variations, all Cystobacterineae CarH homologues tested could replace CarH_Mx_ to regulate P_*crt* Mx_ in *M. xanthus* (Fig. 3B), demonstrating their functional equivalence as well as DNA‐binding plasticity.

### B_12_ ‐dependent photoregulation of carotenogenesis, CarH DNA binding and its abrogation by light or CarS in *Cystobacter*

Among Cystobacterineae, *C. fuscus*, *C. ferrugineus* and *M. boletus* are particularly interesting because, besides CarH, they have all the factors in the CarA pathway except CarA (Fig. 4). Moreover, the *Cystobacter* CarH AdoCbl‐bound form is a dimer (Fig. 3C) whereas CarH_Tt_ or CarH_Bm_ are tetramers, which can have consequences on operator size and/or mode of DNA recognition. We therefore examined how B_12_ affects carotenogenesis in *C. fuscus* and *C. ferrugineus* and how purified *Cystobacter* CarH recognizes operator DNA *in vitro*.

First, we checked if cell culture colour depends on vitamin B_12_ and/or light. Wild‐type *M. xanthus* cell cultures are yellow (due to non‐carotenoid pigments called DKxanthenes) (Meiser *et al*., 2006) in the dark with or without B_12_ in the growth medium because P_*crt* Mx_ is repressed by CarA_Mx_ under both conditions, and also by CarH_Mx_ when B_12_ is available; but a *carA*‐deleted (Δ*carA*) strain is orange in the dark without B_12_, and yellow when B_12_ is present since only CarH_Mx_ can repress P_*crt* Mx_ (Fig. 5B). Both strains turn red in the light, irrespective of the presence or not of B_12_, due to the expression of the *carB* operon as well as of gene *crtIb* (Pérez‐Marín *et al*., 2008; Ortiz‐Guerrero *et al*., 2011). Like the Δ*carA M. xanthus* strain, *C. fuscus* and *C. ferrugineus* were orange without B_12_, yellow or whitish with B_12_ in the dark, and more intensely pigmented under light (Fig. 5B). Consistent with these colour changes, expression of the first gene (*crtE*) in the carotenogenic cluster [estimated by Quantitative Reverse Transcription Polymerase Chain Reaction (qRT‐PCR)] was lower in the dark with B_12_ present, and higher under light (Fig. 5C). Altogether, these data indicate that B_12_ controls light‐induced carotenogenesis in *C. fuscus* and *C. ferrugineus*.

We next tested *in vitro* if and how purified CarH_Cfu_ and CarH_Cfe_ bind to 170‐bp DNA probes containing the 55‐bp segment mentioned earlier. In gel‐shift assays (EMSA), apoCarH_Cfu_ or apoCarH_Cfe_ yielded only a diffuse smear even at high concentrations, suggesting weak non‐specific binding (Fig. S8A). However, in the dark with AdoCbl present, defined retarded bands were observed, which were suggestive of stepwise DNA binding, from a smaller to a larger stable DNA‐protein complex as the protein concentration increased (Fig. 5D and Fig. S8A). Binding was cooperative with an affinity (apparent *K*_D_ ≈ 35 ± 1 nM; Fig. S8B) comparable to the estimates reported for CarH_Tt_ and CarH_Bm_ to their respective operators (Jost *et al*., 2015b; Fernández‐Zapata *et al*., 2018). Exposure to light eliminated the retarded bands, indicating poor DNA binding by the photolyzed CarH_Cfu_ or CarH_Cfe_ monomer (Fig. 5D).

**Fig. 6:**
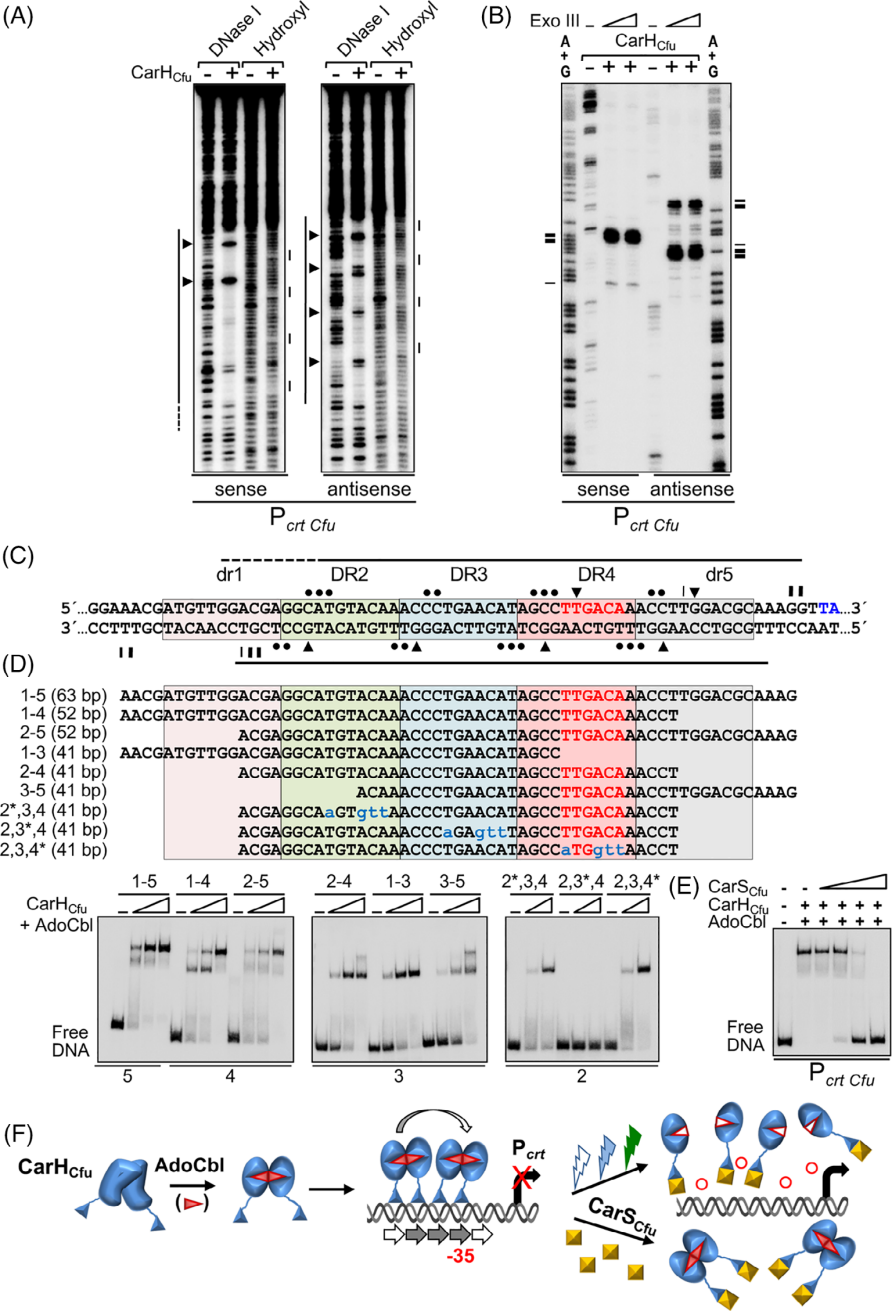
Mapping of the binding site at P_*crt* Cfu_ and mode of action of CarH_Cfu_. A. Representative DNase I and hydroxyl radical footprints upon binding of 100 nM CarH_Cfu_ to the sense and antisense strands of P_*crt* Cfu_. Lines to the left: DNase I footprints; lines to the right: hydroxyl radical footprints; arrowheads: DNase I hypersensitive sites. B. Representative Exo III footprints for CarH_Cfu_ binding to P_*crt* Cfu_. Positions of Exo III arrest: horizontal lines on the side (thicker for stronger arrest). C. Summary of footprint data generated by CarH_Cfu_ binding at P_*crt* Cfu_. DNase I footprints: horizontal lines; DNase I hypersensitive sites: arrowheads; sites protected from hydroxyl radical attack: dots; positions of Exo III arrest: vertical lines (thicker for stronger arrest); **−**35 element: lettered red; **−**10 element (part): blue. DR: tandem 11‐bp direct repeat with the sequence 5 ´‐*nnnn*T*nn*ACA*n*‐3 ´(*n* is any base); dr: 11‐bp direct repeat where the T*nn*ACA motif is not fully conserved. D. Top: Sequences (sense strand) of oligonucleotide probes used, with mutations in blue lowercase. Bottom: Binding to the indicated probes (number of repeats shown below) by CarH_Cfu_ (50, 100 and 200 nM; 100 and 200 nM for mutant DR2‐4 probes). E. Representative EMSA with CarH_Cfu_ (100 nM) and increasing concentrations of its cognate CarS_Cfu_ (100, 200, 400, 800 nM, added after prior incubation of CarH_Cfu_ with P_*crt* Cfu_). DNA binding assays in A–E were performed in the dark with AdoCbl present at five‐fold excess relative to protein. F. Model summarizing CarH_Cfu_ mode of action. ApoCarH_Cfu_ dimers bind to AdoCbl to form active CarH_Cfu_ dimers, which bind cooperatively to an operator at P_*crt* Cfu_ with two overlapping sites, each comprising three tandem DRs (filled grey arrows) and one dr (unfilled arrows). Binding of two CarH_Cfu_ dimers to dr1–DR4 (as shown) or to DR2–dr5 would block the **−**35 promoter region and repress transcription. Light (UV/blue/green) cleaves the AdoCbl Co–C bond to liberate the upper axial ligand (unfilled red circles) provoking disassembly of CarH_Cfu_ dimers to monomers that retain photolyzed AdoCbl (unfilled red triangles) and do not bind DNA. Alternatively, P_*crt* Cfu_ can be derepressed by CarS_Cfu_, which sequesters CarH_Cfu_ to block DNA binding.

We next mapped the site recognized by CarH_Cfu_ at P_*crt* Cfu_ using footprinting analyses. CarH_Cfu_ binding yielded a large DNase I footprint, which coincides well with the segment based on alignment with the CarA_Mx_/CarH_Mx_ operator (Fig. 5A) and which includes part of dr1 and DR2‐dr5. Within the DNase I footprint, four tracts (2–3 nt each) spaced 10–11 nt apart (~1 pitch of double‐stranded B‐DNA) were protected from hydroxyl radical attack on each strand (Fig. 6A and C). Moreover, CarH_Cfu_ binding arrested Exo III at ~4 nt downstream of dr5 (strongly) and DR4 (weakly) in the sense strand, and at ~2–4 nt upstream of DR2 (strongly) and dr1 (weakly) in the antisense strand (Fig. 6B and C). Taken together, the sizes of the DNase I/Exo III footprints and the four evenly spaced hydroxyl radical footprints are consistent with cooperative binding to one face of a DNA site composed of the three tandem DRs plus one dr (dr1‐DR4 or DR2‐dr5, both including the **−**35 promoter region) by two CarH_Cfu_ dimers, each using its two DBDs. Accordingly, CarH_Cfu_ could bind to 52‐bp probes spanning dr1‐DR4 or DR2‐dr5, yielding the two retarded bands observed with the 170‐bp P_*crt* Cfu_ DNA fragment or with a 63‐bp probe spanning all five repeats (Figs. 5D and 6D). By contrast, the upper band normally observed at higher CarH_Cfu_ concentrations was weak for probes with three DRs (2–4) or two DRs and one dr (1–3, 3–5), and absent for probes with two DRs (2*–3–4, 2–3–4*, generated by mutating one of the two outlying DRs of the three DR‐probe), with which a stable retarded band nonetheless continued to be observed (Fig. 6D). Moreover, the CarH_Cfu_ dimer requires the two repeats to be in tandem, since no binding was detected for probe 2–3*–4, with the central DR of the three‐DR probe mutated (Fig. 6D). Stable formation of the larger complex therefore requires at least four repeats and two CarH_Cfu_ dimers, since two DBDs from each dimer would bind to two tandem repeats.

Since CarH_Cfu_ can regulate P_*crt* Mx_ in *M. xanthus*, we probed how it binds to P_*crt* Mx_ *in vitro*. We also tested whether and how CarA_Mx_, a dimer like CarH_Cfu_ but B_12_‐independent, binds to P_*crt* Cfu_, which lacks the palindromes (Fig. 5A) that were suggested as recognized by CarA_Mx_ (López‐Rubio *et al*., 2004). In EMSA, DNase I and Exo III analyses (Fig. S9) both proteins bound in a similar cooperative, stepwise manner to target the same DNA stretch at both P_*crt* Cfu_ or P_*crt* Mx_, despite variations in the DR sequences and their arrangement and the absence of the inverted repeats in P_*crt* Cfu_, suggesting that CarA_Mx_ also recognizes DRs rather than inverted repeats. Differences in some features, like DNase I hypersensitive sites (usually indicative of DNA distortions from protein binding), possibly reflect intrinsic differences between the two proteins, such as AdoCbl dependence only for CarH_Cfu_. Thus, even though CarH_Cfu_ requires AdoCbl for DNA binding and CarA_Mx_ does not, both are dimers with similar modes of DNA recognition. In addition, purified CarS_Cfu_ abolished DNA binding by AdoCbl‐bound CarH_Cfu_ (Fig. 6E), just as CarS_Mx_ abolishes DNA binding by CarA_Mx_. CarH_Cfu_ therefore combines specific AdoCbl‐dependent DNA binding in the dark and direct disruption by light characteristic of its tetrameric homologues CarH_Tt_ and CarH_Bm_, with the cooperative stepwise binding as a dimer to operator DNA and its inhibition by the cognate CarS_Cfu_ that characterizes the B_12_‐independent CarA_Mx_ dimer (Fig. 6F).

### B_12_ ‐dependent photoregulation of carotenogenesis, CarH operator design and DNA binding in Sorangiineae and Nannocystineae

Sorangiineae and Nannocystineae differ from Cystobacterineae, where the majority of carotenogenic genes and *carH* occur as a large syntenic operon with a single promoter region that generally contains three DRs, with at least two in tandem, and two drs. Among Sorangiineae, carotenogenic genes are located at several genomic loci and unlinked to *carH* in *S. amylolyticus* and *L. luteola*, and our search in their putative promoter regions for tandem 11‐bp direct repeats as possible CarH binding sites revealed none in *S. amylolyticus* and only one upstream of *carH* in *L. luteola*, with two tandem DRs and one dr (Fig. S10A). In the remaining Sorangiineae, we could identify one DR flanked by two or three drs in the intergenic segments of the divergently expressed *crtI*‐*carH* and *crtE*‐*crtD* gene pairs, the latter with three DRs in tandem in *S. cellulosum* (P_*crt Sc2*_; Fig. S10A). Similar analyses in Nannocystineae (where *carH* is isolated in *N. exedens* but amidst carotenogenic genes in the others) identified at least one putative CarH binding site with two tandem DRs and two adjacent drs in all except *E. salina*, which has only one DR at both possible sites flanked by 2–3 drs (Fig. S10B). To experimentally test CarH binding to these diverse possible sites, we chose *S. cellulosum* and *H. ochraceum*, which we could grow in liquid cultures and/or plates with or without B_12_, as representative of Sorangiineae and Nannocystineae respectively.

**Fig. 7:**
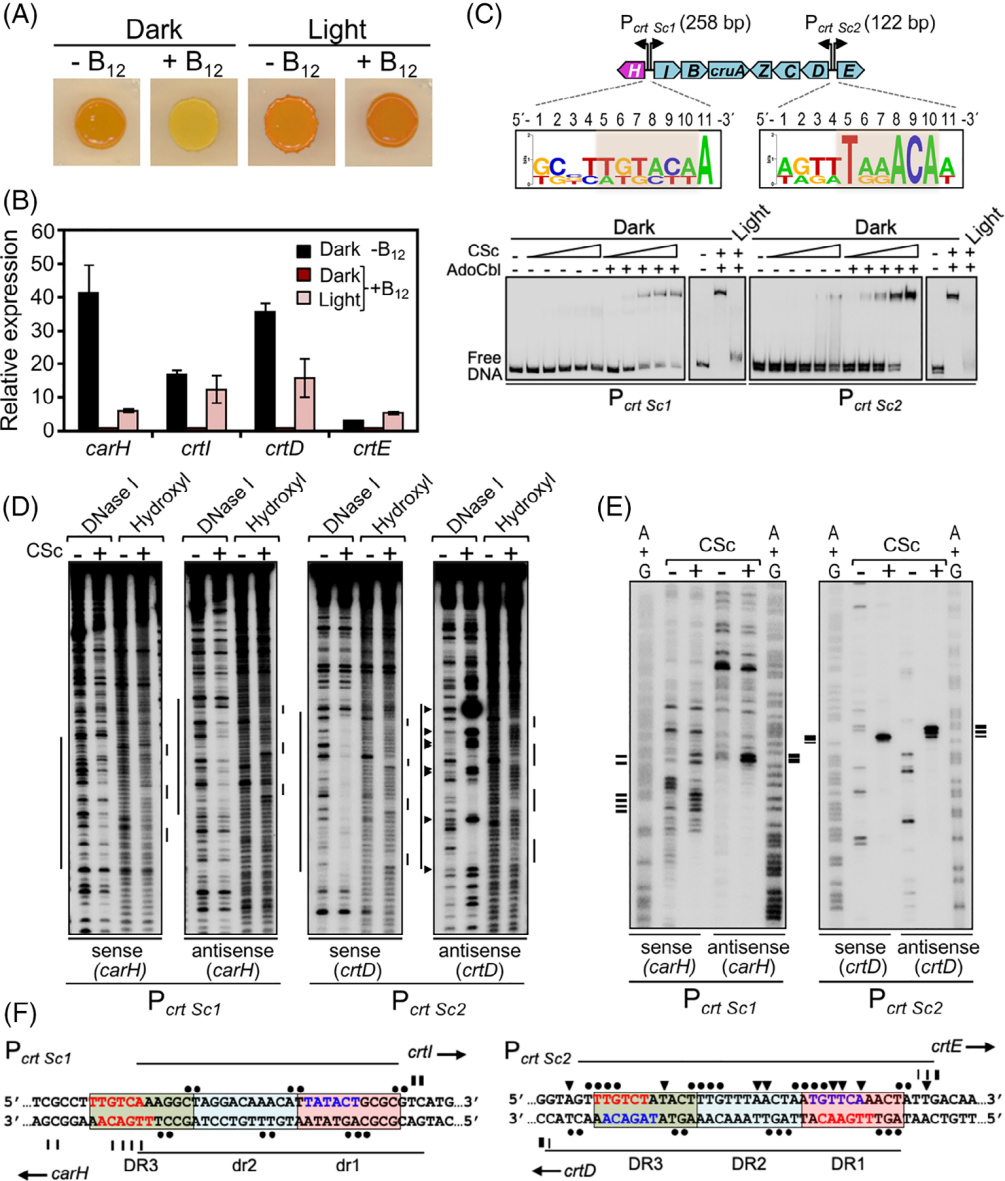
B_12_‐dependent light‐induced carotenogenesis and CarH DNA binding in *S. cellulosum*. A. Colour of *S. cellulosum* cell culture spots on plates with or without vitamin B_12_ incubated for 48 h in the dark or in the light. B. Expression of the indicated gene measured by qRT‐PCR using *rpoD* as the reference gene, and reported relative to the level in the dark with vitamin B_12_ present (mean and standard error from three biological replicates). C. Top: *S. cellulosum* carotenoid synthesis gene cluster with sequence logos (T*nn*ACA segment or equivalent shaded) from the three putative 11‐bp repeats at P_*crt Sc1*_ (dr1, dr2, DR3) and P_*crt Sc2*_ (DR1–DR3). Bottom: Representative EMSA for the binding (in the dark or after 5‐min green light exposure in the presence or absence of AdoCbl) of CSc to P_*crt Sc1*_ (left gel: 60, 120, 240, 480, 960 nM; right gel: 1000 nM CSc) and to P_*crt Sc2*_ (left gel: 10, 20, 40, 80, 120 nM CSc; right gel: 120 nM CSc). D. Representative DNase I and hydroxyl radical footprints on the sense and antisense strands (relative to the indicated gene) for binding of 1000 nM CSc to P_*crt Sc1*_ and 240 nM CSc to P_*crt Sc2*_ (in the dark and presence of AdoCbl). E. Representative Exo III footprint on the sense and antisense strands of probes P_*crt Sc1*_ and P_*crt Sc2*_ upon CSc binding as in D. F. Summary of footprint data at P_*crt Sc1*_ and at P_*crt Sc2*_ upon CSc binding. Proposed promoter elements (red, **−**35 region; blue, **−**10 region) are shown (note overlap of the two divergent promoters). Symbols in D–F are as in Fig. 6A–C respectively.

We found that *S. cellulosum* undergoes B_12_‐ and light‐dependent cell culture colour changes, being orange in the dark without B_12_ or when exposed to light but yellow in the dark with B_12_ present (Fig. 7A), analogous to the Δ*carA M. xanthus* and the two *Cystobacter* strains (Fig. 5B). Moreover, expression of the divergent *crtI–carH* and *crtE–crtD* genes was repressed in the dark in the presence of B_12_ and derepressed upon exposure to light (Fig. 7B), confirming that B_12_ and light modulate expression of carotenoid genes in *S. cellulosum*. We then examined AdoCbl‐dependent binding at the *crtI*–*carH* (P_*crt Sc1*_) and *crtE*–*crtD* (P_*crt Sc2*_) intergenic regions *in vitro*. Because native soluble CarH_Sc_ could not be purified, we resorted to the strategy used previously to test CarH_Mx_ binding to P_*crt* Mx_ *in vitro* (Ortiz‐Guerrero *et al*., 2011). For this, the CarH_Sc_ DBD (and its specific DNA‐binding determinants) and linker were fused to the CarH_Tt_ CBD to generate chimera CSc, which was purifiable. In EMSA, binding of CSc to P_*crt Sc1*_ and P_*crt Sc2*_ was clearly observed only in the dark and with AdoCbl present (Fig. 7C). Consistent with three tandem DRs identified in P_*crt Sc2*_ and only one DR and two drs in P_*crt Sc1*_, CSc exhibited higher affinity for P_*crt Sc2*_, since an eight‐fold lower CSc concentration was sufficient for maximal binding to P_*crt Sc2*_ compared to P_*crt Sc1*_. Moreover, CSc yielded clearer DNase I, hydroxyl and Exo III footprints at P_*crt Sc2*_ than at P_*crt Sc1*_ (Fig. 7D–F). Nonetheless, the three tandem repeats in either probe mapped to the ~34–38 bp DNase I footprints and were delimited by positions where Exo III advance was blocked by CSc. Thus, CSc can bind to a site with three tandem DRs (P_*crt Sc2*_), like CarH_Tt_, but also to one with just one DR and two drs (P_*crt Sc1*_). Putative −35 and/or **−**10 promoter elements that could be identified within the footprinted regions of both probes hint at how CarH_Sc_ binding at a single site in either probe can simultaneously regulate expression from divergent promoters (Fig. 7F).

**Fig. 8:**
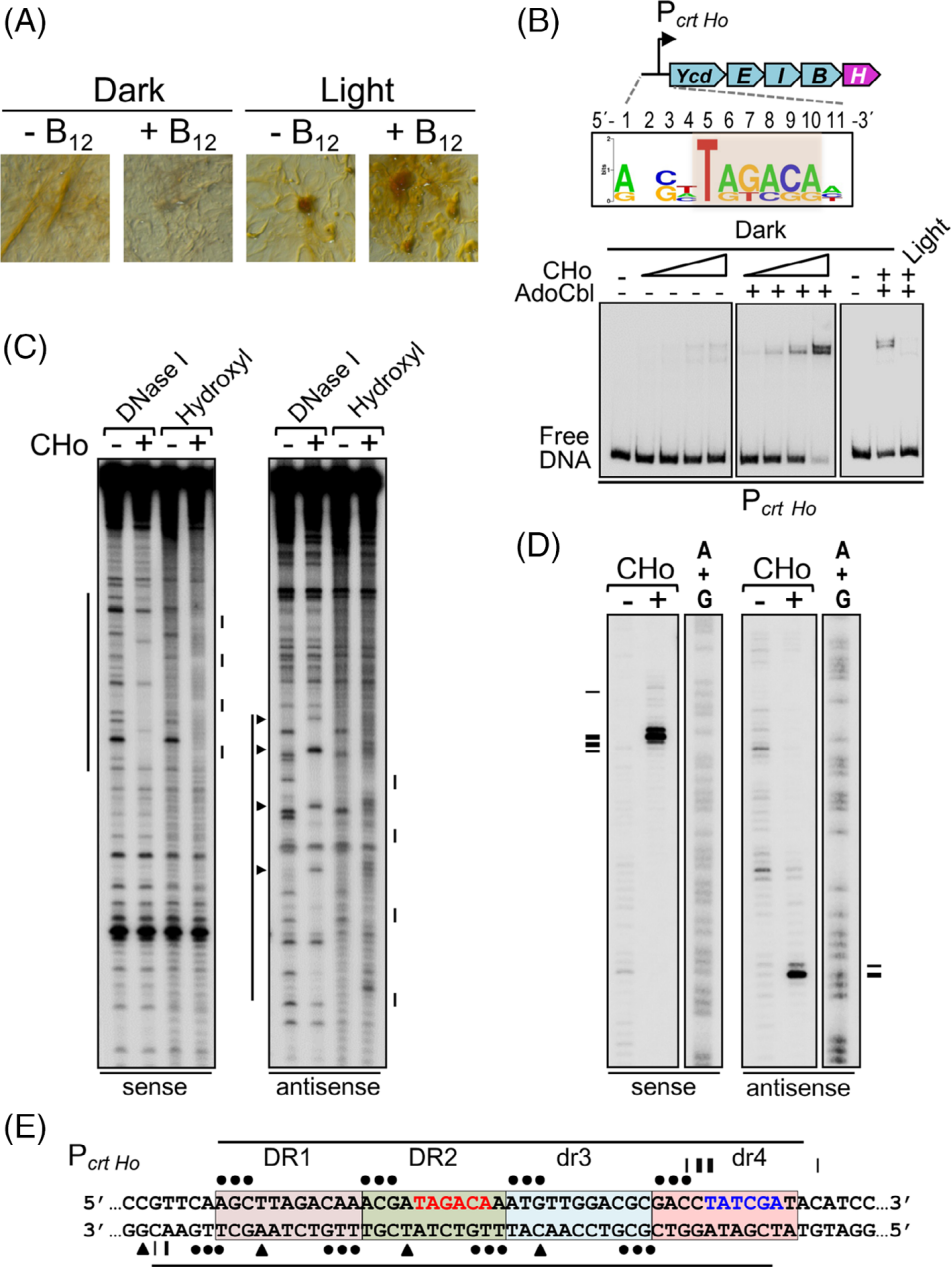
B_12_‐dependent light‐induced carotenogenesis and CarH DNA binding in *H. ochraceum*. A. Colour of *H. ochraceum* cells streaked on plates with or without vitamin B_12_ incubated for 48 h in the dark or in the light. B. Top: *H. ochraceum* carotenoid synthesis gene cluster and sequence logo (T*nn*ACA segment or equivalent shaded) derived from the four repeats at P_*crt Ho*_. Bottom: representative EMSA for the binding of CHo to a 221‐bp P_*crt Ho*_ probe in the presence or absence of AdoCbl in the dark (left and middle gels: 2.5, 5, 10, 15 nM CHo) or after 5‐min green light exposure (right gel: 10 nM CHo). C. Representative DNase I and hydroxyl radical footprinting on the sense and antisense strands of probe P_*crt Ho*_ with or without CHo in the dark and presence of AdoCbl. D. Representative Exo III footprinting of CHo binding to probe P_*crt Ho*_ as in C. E. Summary of footprint data on each strand at P_*crt Ho*_ upon CHo binding. Proposed promoter elements (red, **−**35 region; blue, **−**10 region) are shown. Symbols in C–E are as in Fig. 6A–C respectively.

A similar analysis was carried out for *H. ochraceum*, which was also less pigmented in the dark with B_12_ present than without, or in the light (Fig. 8A). Like CarH_Sc_, CarH_Ho_ was not purifiable as a soluble native protein. We therefore again generated and purified a chimera, CHo, fusing the CarH_Ho_ DBD and linker to the CarH_Tt_ CBD and tested its binding to a 170‐bp probe (P_*crt Ho*_) corresponding to the upstream non‐coding region of the carotenogenic gene cluster containing *carH* (Fig. 8B). CHo could bind to P_*crt Ho*_ only in the dark with AdoCbl present (Fig. 8B) and footprinting analysis mapped the binding site, which overlaps with putative **−**35 and −10 promoter regions, to four tandem 11‐bp repeats comprising two DRs and two drs (Fig. 8C–E). Thus, light and B_12_ also appear to regulate via CarH the expression of carotenogenic gene clusters in Sorangiineae and Nannocystineae, despite their distinct arrangements from that in Cystobacterineae. Moreover, the data reveal, for the first time, that even operators with only one or two DRs (with flanking drs) can be recognized by CarH, highlighting unusual plasticity in DNA recognition and operator design.

## Discussion

Studies on *M. xanthus* light‐induced carotenogenesis have uncovered new paradigms in bacterial light sensing, signalling and response, and led to the discoveries of widely distributed protein families with novel functions. Central players in the genetic circuit underlying this *M. xanthus* response to light are two transcriptional factors with similar domain architectures and DNA target sites, CarH_Mx_ and CarA_Mx_, which control the expression of the same genes by distinct mechanisms. While an AdoCbl chromophore directly senses light to allosterically modulate CarH repressor activity, regulation by CarA requires the antirepressor CarS, whose light‐induced expression relies on a multifactorial pathway that additionally includes CarF, CarQ, CarR, CarD and CarG. Our present study on the conservation of these elements for photoinduced carotenogenesis across diverse myxobacteria provides molecular and evolutionary insights into these factors and establishes that B_12_‐dependent photoregulation of carotenogenesis pervades in this major class of social and predatory soil and marine bacteria.

Our sequence analysis with experimental validation assigned CarH homologues in all the myxobacteria examined, except in two species with reduced genomes, *A. dehalogenans* and *V. incomptus*. Both of these also lack genes for carotenoid synthesis and probably rely on ROS detoxifying enzymes and/or light‐avoidance mechanisms to combat photooxidative stress. The majority have *carH* in the genomic neighbourhood of genes for carotenogenesis, which occur as a large syntenic block in Cystobacterineae and as smaller syntenic blocks in Sorangiineae or Nannocystineae. Genomic proximity of *carH* to carotenogenic genes points to functional, presumably evolutionary, links between them that may be related to CarH's ability to sense light and elicit the photoprotective response over a broader spectral range (UV to green) than most other known photoreceptors. In contrast to CarH, our analysis revealed CarA only in suborder Cystobacterineae, always with CarH, and invariably encoded by the gene immediately upstream of *carH* in the carotenogenesis gene cluster characteristic of this suborder. That *carA* and *carH* exist in tandem and their close phylogenetic relationship suggests that their co‐occurrence is likely due to gene duplication. Gene duplication and divergence is a major mechanism for the evolution of new genes or paralogues with overlapping or non‐overlapping functions and has been proposed as a key contributor to genomic expansion and sensory complexity in myxobacteria (Goldman *et al*., 2006; Schneiker *et al*., 2007). Tandemly duplicated genes, usually arising from recombination‐based mechanisms, are often restricted to closely related species, tend to share similar functions and regulatory elements, and typically contribute to adaptation and response to environmental stimuli and species‐specific traits (Simon *et al*., 2015; Chapal *et al*., 2019; Kuzmin *et al*., 2020). All of these apply to the CarH–CarA pair. Given the ancient prebiotic origin proposed for B_12_ (Roth *et al*., 1996; Monteverde *et al*., 2017), the phylogeny, and far greater prevalence of CarH over CarA, it is likely that an ancestral AdoCbl‐dependent CarH duplicated and diverged to create a B_12_‐independent CarA. The distribution of *carH* and *carA* genes in the myxobacterial genomes analysed in this study suggests that such a gene duplication could have occurred in the ancestor of the *Myxococcus‐Archangium* branch or, alternatively, in an even earlier Cystobacterineae predecessor, with subsequent gene loss resulting in just one copy in *C. ferrugineus*, *C. fuscus* and *M. boletus*, and none in *A. dehalogenans* and *V. incomptus*.

Transition from CarH to CarA entailed loss of the Wx_9_EH motif, crucial for AdoCbl‐specific binding, oligomerization and DNA binding of CarH, together with changes to ensure that CarA forms stable dimers independently of B_12_. Most remarkably, photosensory regulation by CarA has required coevolution of various singular proteins, some eukaryotic‐like, and a signature metazoan lipid. Myxobacteria are the only strictly aerobic bacteria known to have the oxygen‐dependent desaturase (CarF) for plasmalogen biosynthesis whose equivalent, despite the enormous evolutionary distance, is specifically conserved in metazoa (Gallego‐García *et al*., 2019). The existence in myxobacteria of factors considered typically eukaryotic point to tangible evolutionary links to eukaryotes and possible horizontal gene transfer events that provide supporting evidence for the Syntrophy hypothesis, which posits an ancient myxobacterium as crucial in eukaryogenesis (López‐García and Moreira, 2020; Hoshino and Gaucher, 2021). Thus, steroid biosynthesis has been proposed to have first evolved in an early myxobacterium in response to the rise of oxygen and later horizontally transferred to an early eukaryote, because myxobacteria form the select set of aerobic bacteria to have both the steroid and its isoprenoid precursor (MVA) biosynthesis pathways homologous to those in eukaryotes (Hoshino and Gaucher, 2021). It is tempting to speculate that this also extends to the oxygen‐based biosynthesis of plasmalogens, given the existence of CarF in myxobacteria and in metazoans, and its absence in nearly all other bacteria. However, while eukaryotic‐type steroid biosynthesis enzymes appear across all three myxobacterial suborders (Hoshino and Gaucher, 2021), we found CarF almost entirely in Cystobacterineae. Curiously, besides the gene for HMGR that is part of an operon with various other MVA pathway genes, the large syntenic block of carotenogenic genes in some Cystobacterineae includes a second copy of a gene for HMGR. Since HMGR is generally a rate‐limiting enzyme in the MVA pathway, its photoinduced co‐expression with carotenogenic enzymes possibly ensures ample supply of the earliest precursors (IPP/DMAPP) of carotenoids for light‐induced production.

CarR orthologs also appear to have evolved solely in Cystobacterineae to act in concert with plasmalogens and ^1^O_2_ in a photoprotective stress response. They diverge significantly in sequence from other DUF1109 anti‐σ but may nonetheless retain a role in responses to metals, as observed for CarR_Mx_ in the copper response. Moreover, CarR has had to co‐evolve with CarQ to recognize it specifically from among the numerous ECF‐σ factors typically found in myxobacteria. CarS homologues are yet another class of proteins found exclusively in Cystobacterineae. CarS is a small, taxonomically restricted protein acting in a stress response and thus has features typically ascribed to a *de novo* gene product (Schlotterer, 2015; Van Oss and Carvunis, 2019). Also crucial in the CarA pathway is the highly unique CarD–CarG complex, formed by two proteins encoded by adjacent genes that always co‐exist in every (and only in) Cystobacterineae analysed here. In CarD, the fusion of an N‐terminal RNAP‐interacting module of exclusively bacterial origin with a C‐terminal DNA‐binding domain that resembles eukaryotic HMGA or histone H1 is intriguing at the evolutionary and functional levels. It has been proposed that the origins of the characteristic K/A/P‐rich H1 C‐terminal region can be traced to eubacteria, from where it was acquired by horizontal gene transfer and evolved in eukaryotes (Kasinsky *et al*., 2001). If so, an ancestral CarD could have arisen from fusion of two bacterial modules, one of which was a C‐terminal K/A/P‐rich H1‐like region as in *A. dehalogenans*/*V. incomptus*, from which the remaining Cystobacterineae acquired the HMGA AT‐hooks by convergent evolution. Two independent fusion events, one to an H1‐like region and another to an HMGA‐like region, would be less probable. Since CarD always functions as a single unit with CarG, the two proteins must have co‐evolved. Horizontal gene transfer followed by functional divergence may have led to the emergence of CarG, as it lacks any protease activity yet has a zinc‐binding metalloprotease motif of the type typically found only in archaeal and metazoan metzincins.

Strikingly, *Cystobacter/Melittangium* species in Cystobacterineae lack CarA despite having all other known factors of this pathway. The single *Cystobacter* CarH/A homologue, our data show, is an AdoCbl‐based CarH photoreceptor that transitions from an inactive dimeric apo form to active AdoCbl‐bound dimers, which become inactive monomers upon photolysis. The contrast with the oligomeric transitions of the only other well‐studied CarH homologues, CarH_Tt_ (monomer–tetramer–monomer) and CarH_Bm_ (tetrameric molten globule‐tetramer–dimer) highlights the oligomerization plasticity of these photoreceptors. Oligomerization confers properties crucial for protein function, like cooperative ligand binding, allosteric regulation and stability and changes in oligomerization through evolution usually enable new regulatory modes. But how a given multimer and its functions arise is poorly understood. Extant oxygen‐binding haemoglobin tetramers, with their striking cooperativity and regulatory modes, may have evolved by successive gene duplication‐divergence events through surprisingly few mutations from an ancestral dimeric precursor, which itself arose from an ancient monomer (Pillai *et al*., 2020). Single mutations at the interface between the two head‐to‐tail dimers that constitute the CarH_Tt_ tetramer suffice to shift the tetramer to a native dimer, which retains AdoCbl‐dependent DNA binding, but with altered affinity and cooperativity (Jost *et al*., 2015b); CarH_Tt_ tetramerization traces a kinetic pathway from monomer to dimer to tetramer (Camacho *et al*., 2021). Hence, few genetic changes may underlie the shift between the AdoCbl‐dependent *Cystobacter* CarH dimer and its tetrameric homologues.

We found that despite the distinct oligomerization, the *Cystobacter* CarH dimer emulates its tetramer homologues in its AdoCbl‐dependent binding to one face of a DNA region comprising 11‐bp tandem direct repeats. It does so by cooperative binding as two dimers, effectively a tetramer, to two overlapping sites, each composed of three tandem DRs and one dr. Whereas each CarH_Cfu_ dimer requires the two repeats to be in tandem, in the case of the CarH_Tt_ tetramer one head‐to‐tail dimer interacts with two non‐contiguous DRs (DR1 and DR3) and the other contacts the central DR (DR2) using only one of its two DBDs (Jost *et al*., 2015b). Furthermore, CarH_Cfu_ and CarA_Mx_ recognize each other's operators *in vitro* and employ a similar DNA binding mode, regardless of the variations between their natural sites and the requirement for AdoCbl by CarH_Cfu_ but not by CarA_Mx_; CarH_Cfu_ can direct AdoCbl‐based regulation of P_*crt* Mx_ *in vivo*, emphasizing the evolutionary relationship between the two proteins and their operators. Indeed, in every CarH‐containing Cystobacterineae analysed we discerned counterparts to the *M. xanthus* and *Cystobacter* operators in size and sequence, generally with three DRs (and two drs) and an identical **−**35 promoter region embedded in one of them, such that binding of CarH would block access to RNAP. In Sorangiineae and Nannocystineae, CarH operators are less like those in Cystobacterineae. Most species appear to have two operators that differ among themselves, each expected to control two divergent promoters, given the overlap with putative promoter regions. Thus, two CarH operators were experimentally mapped in *S. cellulosum*, one comprising three tandem DRs and the other just one DR and two drs, while the single operator mapped in *H. ochraceum* has two DRs and two drs. Even though CarH operators across the three suborders are quite varied, CarH homologues from the three myxobacterial suborders could functionally replace CarH_Mx_ for AdoCbl‐dependent photoregulation of P_*crt* Mx_ in *M. xanthus*. This highlights an unusual DNA‐binding plasticity of these homologues, possibly providing cells a strategy to fine–tune affinities and operator occupancies for optimal regulation *in vivo*.

The B_12_‐independent CarA pathway, absent in both Sorangiineae and Nannocystineae, affords an alternative mechanism to the B_12_‐dependent CarH one in Cystobacterineae. Parallel mechanisms usually confer a fitness advantage by boosting response efficiency and control, and enable adaptive evolution through cooperation and functional divergence to bestow benefits not gained from just a single mechanism (Chapal *et al*., 2019; Kuzmin *et al*., 2020). Direct light‐sensing and regulation by AdoCbl‐CarH ensures a very rapid and efficient response. However, myxobacteria cannot perform the expensive *de novo* biosynthesis of the complex AdoCbl cofactor, which requires as many as 30 steps, because they do not have all the necessary enzymes (Shelton *et al*., 2019). They consequently must depend on their environmental niche to acquire B_12_, a frequently transacted resource in microbial consortia (Sokolovskaya *et al*., 2020), by preying on microorganisms that can supply B_12_ or a suitable precursor, or by importing these directly from the milieu. A need to cope with limited B_12_ probably drove the emergence of the CarA pathway in myxobacteria, which allows them to still regulate the photoprotective response when B_12_ is unavailable. An additional adaptative advantage conferred by this pathway is its possible implication in other independent or overlapping responses, as illustrated by its role in the response to copper. Even without CarA, presence of the other factors in the B_12_‐independent pathway would expand the range of responses beyond that to light. Moreover, given that CarQ is an alternative ECF‐σ factor, its regulon may extend beyond genes for carotenoid synthesis or their specific regulators (our unpublished data point to various genes against oxidative stress). This may explain the occurrence of these other B_12_‐independent pathway factors even in the absence of CarA, as in *Cystobacter*, and opens the possibility that these other factors may have emerged prior to the appearance of CarA from an ancestral B_12_‐dependent CarH photoreceptor.

## Experimental procedures

### Bacterial strains, growth conditions and complementation analysis

Bacterial strains, their source and plasmids used in this study are listed in Table S3. *E. coli* strain DH5α used for plasmid constructs, BL21(DE3) for protein overexpression, and BTH101 (*cya* ^*−*^) for BACTH analysis, were grown in LB (Luria–Bertani) liquid medium or LB‐agar (1.5%) plates at 37°C or 25°C/18°C for inducing His_6_‐tagged protein overexpression. *M. xanthus* was grown at 33°C in CTT medium (10 g L^−1^ Bacto‐casitone, 10 mM Tris‐HCl pH 7.6, 1 mM KH_2_PO_4_, 8 mM MgSO_4_), as reported previously (Ortiz‐Guerrero *et al*., 2011). *S. cellulosum* was grown at 33°C in M medium (Müller and Gerth, 2006). *C. fuscus*, *C. ferrugineus* and *M. boletus* were grown at 30°C in 3 g L^−1^ Bacto‐casitone, 1 g L^−1^ glucose, pH 7.2, and *H. ochraceum* at 30°C on CY 1:5 plates (0.6 g L^−1^ Bacto‐casitone, 0.2 g L^−1^ yeast extract, 0.27 g L^−1^ CaCl_2_·2H_2_O, 21.1 g L^−1^ NaCl, 0.6 g L^−1^ KCl, 3.6 g L^−1^ MgCl_2_·6H_2_O, 0.09 g L^−1^ NaHCO_3_, 2.6 g L^−1^ MgSO_4_.7H_2_O, 15 g L^−1^ Difco agar, pH 7.2). Media were supplemented with 1 μg ml^−1^ of vitamin B_12_ (which is converted intracellularly to AdoCbl) or AdoCbl and, as required, grown under light using three 18‐W fluorescent lamps (10 Wm^−2^). Genes for CarA_Av_, CarH_Av_, CarH_Ho_, CarH_Pp_, CSc and Cho were synthesized (GenScript). The others examined were PCR‐amplified from genomic DNA purchased from DSMZ, Germany (*C. coralloides*), kindly provided by Prof. Rolf Müller (*M. fulvus*), or isolated using Wizard genomic DNA purification kit (Promega) from cells (*C. fuscus*, *C. ferrugineus* and *M. boletus*) grown as described above. For complementation analysis, the gene of interest was cloned into plasmid pMR3679 (Km^R^), which allows conditional expression from a vanillate‐inducible promoter, tunable by inducer concentration (Iniesta *et al*., 2012). The construct, verified by sequencing, was electroporated into *M. xanthus* strain MR2649, which harbours a Tn5‐*lac‐132* insertion in the *carB* operon to serve as a P_*crt* Mx_::*lacZ* reporter probe and lacks endogenous CarH, CarA, CarS and to allow controlled supply of exogenous AdoCbl, PduO (Ortiz‐Guerrero *et al*., 2011). Transformants with plasmid integration at a heterologous site by homologous recombination were selected using the Km^R^ marker. *M. xanthus* cells were grown to exponential phase (OD_600_ ~ 0.8) in the dark in liquid CTT media with vanillate (2–500 μM depending on the CarH homologue), antibiotics and 1 μg ml^−1^ of AdoCbl as required, diluted into fresh medium of the same composition and grown to OD_600_ ~ 0.8, then split into two cultures, one for growth in the dark and the other under light for 8 h. Cells were spotted (5 μl) on plates of the same composition and grown under the same conditions, or collected for P_*crt* M*x*_::*lacZ* β‐galactosidase specific activity measurements (in nmol o‐nitrophenyl‐β‐d‐galactoside hydrolyzed/min mg^−1^ protein reported as the mean and standard error of three biological replicates) in a SpectraMax 340 microtitre plate reader (Molecular Devices), as described previously (Ortiz‐Guerrero *et al*., 2011).

### Genome and phylogenetic analysis

Specific *M. xanthus* proteins were used as queries in BLASTP analysis of myxobacterial genomes at the JGI integrated microbial genomes (IMG) resource (https://jgi.doe.gov/data-and-tools/img) to identify myxobacterial orthologs in the light response and in the MVA pathway as well as their genome contexts. The default E‐value cut‐off (1E‐5) was used in all the analyses (Table S2 lists percent identities, E‐values and other details). Among those retrieved with CarA_Mx_ or CarH_Mx_ as queries against each genome, the best hits were assigned as true homologues, and all had E‐values ≤ 1E‐17, percent identities ≥ 29%, contained an N‐terminal MerR family DBD and a C‐terminal CBD, and were in the 286–330 amino acid residue size range. Myxobacterial carotenoid, isoprenoid and cobalamin biosynthetic pathways were additionally analysed using the KEGG pathways database (https://www.genome.jp/kegg), BLASTP (https://blast.ncbi.nlm.nih.gov/Blast.cgi), and Conserved Domains search tool (https://www.ncbi.nlm.nih.gov/Structure/cdd/wrpsb.cgi). Protein sequence identities were visualized with Heatmapper (http://www.heatmapper.ca/). Maximum likelihood phylogenetic trees were generated using MEGA7 (Molecular Evolutionary Genetics Analysis version 7.0; https://www.megasoftware.net), the JTT (Jones–Taylor–Thornton) substitution model and 200 bootstrap iterations after alignment of CarH/A homologues with MUSCLE (in MEGA7) or of 16S rRNA sequences for Myxococcales species with MAFFT (version 7.0; https://www.ebi.ac.uk/Tools/msa). Trees were visualized and displayed using FigTree v1.4.4 (http://tree.bio.ed.ac.uk/software/figtree) and iTOL (version 5; https://itol.embl.de). Putative CarH DNA‐binding sites were identified by visual inspection of regions upstream of candidate target genes; and bioinformatic searches between positions **−**150 and + 25 relative to the annotated translation start codon for sites conforming to three 11‐bp direct repeats with an N_4_‐T‐N_3_‐C[C][G] consensus sequence (N is any base), and at least one N_4_‐T‐N_2_‐RCR[G] repeat (R is A or G) using R packages: Biostrings (https://bioconductor.org/packages/Biostrings), IRanges (Lawrence *et al*., 2013), dyplr (https://CRAN.R-project.org/package=dplyr) and SeqinR (Charif and Lobry, 2007). Genomic data downloaded from Ensembl Bacteria database in FASTA and GFF3 formats were appropriately converted for data handling. WebLogo (https://weblogo.berkeley.edu/logo.cgi) was used to generate logos from sequences aligned with MUSCLE (gaps removed in MEGA7) or ClustalOmega (https://www.ebi.ac.uk/Tools/msa).

### Quantitative RT‐PCR

RNA was isolated from 5 ml of myxobacterial cultures grown in liquid media with or without 1 μg ml^−1^ of vitamin B_12_ in the dark or under light, reverse transcribed and analysed by qRT‐PCR, as described in detail previously (Fernández‐Zapata *et al*., 2018). Primers used (Table S4) were designed using Primer Express 3.0 software to amplify an ~ 50–150 bp region within each transcript. Target gene expression is reported relative to *rpoD* as reference gene and normalized to the level of expression of the gene in the dark with vitamin B_12_ present, as the mean and standard error for three biological replicates.

### BACTH analysis

Two‐hybrid analysis of light and B_12_‐dependent oligomerization employed the BACTH system (based on protein fusion to the T25 and T18 fragments of *Bordetella pertussis* adenylate cyclase), as described previously (Ortiz‐Guerrero *et al*., 2011). Self‐interaction was inferred from the blue colour of cell spots on 40 μg ml^−1^ of X‐Gal (5‐bromo‐4‐chloro‐3‐indolyl‐β‐d‐galactoside)‐LB plates with or without 1 μg ml^−1^ of vitamin B_12_ grown in the dark or under light and, as controls, CarH_Mx_ and CarA_Mx_. Pairs with just one fusion protein were used as negative controls and no autoactivation was observed, as shown in Fig. S3 for CarH_Cfu_, CarH_Cfe_ and CarH_Mb_.

### Protein purification and characterization

His_6_‐tagged proteins were overexpressed in *E. coli* BL21(DE3) using pET15b constructs, purified and stored as reported before (López‐Rubio *et al*., 2002; Ortiz‐Guerrero *et al*., 2011). Native apoproteins purified in a final size‐exclusion chromatography step (in 150 mM NaCl, 50 mM phosphate buffer, pH 7.5, 2 mM β‐mercaptoethanol) were concentrated and their identities checked by SDS‐PAGE and electrospray ionization‐time‐of flight mass spectrometry (Agilent 1100 Series HPLC, Supelco Discovery BioWide Pore C5 2.1 × 10 cm, 5 μm HPLC column, Ion Trap XCT Plus mass spectrometer with an electrospray interface; mass spectra in the positive ion mode, *m/z* range 100 to 2200 for samples separated using a linear 4%–90% water/acetonitrile/formic acid gradient). AdoCbl was always handled under dim light and checked by UV–visible (UV–Vis) spectroscopy. Protein concentrations were determined from absorbance at 280 nm in a Cary 60 spectrophotometer, using molar extinction coefficients ε_280_ (in M^−1^ cm^−1^) of 37,930, 36,440, 30,480, 32,430 and 30,940 for CarH_Cfu_, CarH_Cfe_, CarS_Cfu_, CSc and Cho, respectively, and the Bio‐Rad protein assay kit.

### UV–Vis absorption spectroscopy and analytical size exclusion chromatography

UV–visible spectra for AdoCbl‐bound CarH_Cfu_ and CarH_Cfe_ photoconversion were recorded for samples in the dark and with stepwise green light irradiation (522 nm, 10 Wm^−2^, from a computer‐controlled LED array) from 1 to 450 s. Analytical size‐exclusion chromatography to estimate the apparent molecular mass (*M*_r_) was performed in an AKTA HPLC unit using a Superdex200 HR 10/30 column and calibrated yielding log *M*_r_ = 8.1537–0.2316 *V*_*e*_, where *V*_*e*_ is the elution volume. Pure protein (100 μl, 50–100 μM), alone or after 15‐min incubation with five‐fold molar excess of AdoCbl was injected into the column in the dark or after 5‐min green light exposure. Elution (0.4 ml min^−1^ flow) was tracked by absorbance at 280, 361 and 522 nm, the peaks collected and analysed for *M*_r_, and by UV–Vis spectroscopy and SDS‐PAGE.

### DNA binding assays

DNA probes > 100 bp were obtained by PCR using appropriate primers, one of them ^32^P‐labelled at the 5′‐end with T4 polynucleotide kinase (Takara). For DNA probes ≤ 63 bp, complementary HPLC‐purified synthetic oligonucleotides (Merck), one ^32^P‐labelled at the 5′‐end and the other unlabeled, were mixed at a 1:2 ratio, incubated at 100°C for 2 min and hybridized by slow cooling. EMSA was carried out using 20 μl of samples containing the DNA probe (1.2 nM, ~13,000 cpm) and protein at specific concentrations, with or without a 5‐fold excess of AdoCbl relative to protein in 0.1 M KCl, 10 mM MgCl_2_, 0.025 M Tris‐HCl (pH 8), 1 mM dithiothreitol, 10% glycerol, 200 ng μl^−1^ of bovine serum albumin and 1 μg sheared salmon sperm DNA as non‐specific competitor. Samples were incubated at 37°C for 30 min and loaded onto a 6% non‐denaturing polyacrylamide gel (37.5:1 acrylamide: bisacrylamide) pre‐run for 30 min in 0.5× TBE buffer (45 mM Tris base, 45 mM boric acid, 1 mM EDTA). All steps were carried out in the dark (under dim light) except when, prior to electrophoresis, samples were exposed for 5 min to green light. Electrophoresis was carried out in the dark for 1.5 h at 200 V and 10°C. Gels were vacuum‐dried and scanned using Personal Molecular Imager™ (PMI™) FX with Quantity One 4.4 software (BioRad). The fraction bound, estimated from band intensities (quantified using ImageJ, NIH) for the free DNA, was fit to a 3‐parameter Hill equation using SigmaPlot (Systat Software) to estimate the apparent equilibrium dissociation constant (*K*_D_, the protein concentration at half‐maximal binding) and Hill coefficient. For DNase I footprinting, 20 μl of samples of a DNA probe (∼20,000 cpm with either the sense or the antisense strand 5′‐end ^32^P‐labelled) and protein with AdoCbl at a 5‐fold excess in EMSA buffer lacking glycerol were incubated for 30 min at 37°C, then treated with 0.07 units of DNase I (Promega) at 37°C for 2 min and quenched with 25 mM EDTA, all in the dark. DNA was ethanol‐precipitated, washed twice with 70% ethanol and dried. For hydroxyl radical footprinting, samples were prepared as for DNase I footprinting but without MgCl_2_, and then treated for 4 min at 25°C with 2 μl each of freshly prepared Fe(II)‐EDTA solution (1 mM ammonium iron (II) sulfate, 2 mM EDTA), 0.01 M sodium ascorbate and 0.6% hydrogen peroxide (Merck). The reaction was stopped with 2 μl each of 0.1 M thiourea and 0.5 M EDTA (pH 8), and DNA precipitated and dried as before. For Exo III (3′ → 5′ exodeoxyribonuclease; ThermoFisher Scientific) footprinting, 20 μl samples of the DNA probe 5′‐end ^32^P‐labelled on a specific strand were mixed with protein and a 5‐fold excess of AdoCbl, incubated in DNase I buffer for 30 min in the dark, then treated with 2.5–5 U μl^−1^ of Exo III for 15 min at 37°C, quenched with 20 mM EDTA, DNA precipitated and dried. Each dried DNA sample for footprinting assays was resuspended in 5 μl of formamide loading buffer, incubated at 95°C for 3 min, and loaded onto 6% polyacrylamide‐8 M urea sequencing gels. After electrophoresis (2–3 h, 50 mA/60 W/1.90 kV), the gels were vacuum‐dried and scanned using PMI™ (BioRad). A + G chemical sequencing ladders were used to map the footprints.

## Supporting information


**Appendix** S1: Supporting Information

**Table S2**: Genome and gene product details for myxobacteria examined in this study
