# Supplementary material for: Coenzyme B12 ‐dependent and independent photoregulation of carotenogenesis across Myxococcales
Source: Environ Microbiol. 2022 Jan 27;24(4):1865–86. doi: 10.1111/1462-2920.15895 (PMC9304148; doi:10.1111/1462-2920.15895)
Supplement: Supplementary file 1 — Appendix S1: Supporting Information [file EMI-24-1865-s002.pdf]

Supporting Information

Coenzyme B_{12}-dependent and independent photoregulation of carotenogenesis across Myxococcales

Ricardo Pérez-Castaño[1], Eva Bastida-Martínez[1], Jesús Fernández-Zapata[2], María del Carmen Polanco[1], María Luisa Galbis-Martínez[1], Antonio A. Iniesta[1], Marta Fontes[1], S. Padmanabhan[2]*, Montserrat Elías-Arnanz[1]*

[1]Departamento de Genética y Microbiología, Área de Genética (Unidad Asociada al IQFR-CSIC), Facultad de Biología, Universidad de Murcia, 30100 Murcia.

[2]Instituto de Química Física "Rocasolano", Consejo Superior de Investigaciones Científicas, 28006, Madrid.

*Corresponding authors. Email: melias@um.es (M.E.-A.); padhu@iqfr.csic.es (S.P.)

This file includes:

Table S1. Characteristics of myxobacteria used for genome analysis
Table S3. Bacterial strains and plasmids used in this work
Table S4. Primers used for qRT-PCR
Figures S1 to S10
Supporting Information References

Table S1. Characteristics of myxobacteria used for genome analysis

Species*	Abbreviation	Strain	Genome (Mb)	(%GC)	Habitat, growth
**Cystobacterineae**					
Anaeromyxobacter dehalogenans	*Ad*	DSM 21875	5.03	74.7	Soil, an/aerobe
Archangium gephyra	*Ag*	DSM 2261	12.49	69.4	Soil, aerobe
Archangium violaceum	*Av*	DSM 14727	12.54	68.9	Soil, aerobe
Corallococcus coralloides	*Cc*	DSM 2259	10.08	69.9	Soil, aerobe
Cystobacter ferrugineus	*Cfe*	DSM 52764	12.05	68.5	Soil, aerobe
Cystobacter fuscus	*Cfu*	DSM 2262	12.28	68.5	Soil, aerobe
Hyalangium minutum	*Hm*	DSM 14724	11.19	68.0	Soil, aerobe
Melittangium boletus	*Mb*	DSM 14713	9.91	68.4	Soil, aerobe
Myxococcus fulvus	*Mf*	DSM 16525	10.82	70.0	Soil, aerobe
Myxococcus hansupus	*Mh*	DSM 436	9.49	69.2	Soil, aerobe
Myxococcus macrosporus	*Mm*	DSM 14697	8.97	70.6	Soil, aerobe
Myxococcus stipitatus	*Ms*	DSM 14675	10.35	69.2	Soil, aerobe
Myxococcus virescens	*Mv*	DSM 2260	9.24	69.2	Soil, aerobe
Myxococcus xanthus	*Mx*	DK1622	9.14	68.9	Soil, aerobe
Stigmatella aurantiaca	*Sa*	DW4/3-1	10.26	67.5	Soil, aerobe
Stigmatella erecta	*Se*	DSM 16858	9.22	69.4	Soil, aerobe
Vulgatibacter incomptus	*Vi*	DSM 27710	4.35	68.9	Soil, aerobe
**Sorangiineae**					
Chondromyces apiculatus	*Cha*	DSM 436	11.58	70.3	Soil, aerobe
Chondromyces crocatus	*Chc*	DSM 14714	11.39	68.7	Soil, aerobe
Labilithrix luteola	*Ll*	DSM 27648	12.19	66.1	Soil, aerobe
Minicystis rosea	*Mr*	DSM 24000	16.04	69.1	Soil, aerobe
Sandaracinus amylolyticus	*Sam*	DSM 53668	10.33	72.0	Soil, aerobe
Sorangium cellulosum	*Sc*	DSM 53796	13.03	71.4	Soil, aerobe
**Nannocystineae**					
Enhygromyxa salina	*Es*	DSM 15201	10.6	68.2	Aquatic, aerobe
Haliangium ochraceum	*Ho*	DSM 14365	9.45	69.5	Aquatic, aerobe
Nannocystis exedens	*Ne*	DSM 71	12.06	72.1	Soil, aerobe
Plesiocystis pacifica	*Pp*	DSM 14875	10.59	70.7	Aquatic, aerobe

* None have the complete set of B_{12} biosynthesis genes based on (Shelton, et al. 2019) and our analysis.

Table S3. Bacterial strains and plasmids used in this work*

Strain or plasmid	Description	Source/Reference
E. coli		
DH5α	F^-φ80*lacZ* ΔM15 Δ(*lacZYA-argF*)U169 *recA1 endA1* hsdR17(r_K^-, m_K^+) *phoA supE44* λ^-*thi-1 gyrA96 relA1*	(Hanahan 1983)
BL21(DE3)	F^-*ompT hsdS*$_B$ (r_B^-, m_B^-) *gal dcm* (DE3)	Novagen
JM109	*endA1*, *recA1*, *gyrA96*, *thi*, *hsdR17* (r_k^-, m_k^+), *relA1*, *supE44*, Δ(*lac-proAB*), [F′ *traD36*, *proAB*, *laqI*^{q}ZΔM15]	(Yanisch-Perron, et al. 1985)
BTH101	F^-*cya-99 alaD139 galE15 galK16 rpsL1 hsdR2 mcrA1 mcrB1*	(Karimova, et al. 2000)
Myxobacteria		
C. coralloides	DSM 2259	DSMZ (Germany)
C. ferrugineus	DSM 52764	DSMZ (Germany)
C. fuscus	DSM 2262	DSMZ (Germany)
H. ochraceum	DSM 14365	DSMZ (Germany)
M. fulvus	Mxf65	Prof. Rolf Müller
M. xanthus	DK1050	(Ruiz-Vázquez and Murillo 1984)
P. pacifica	DSM 14875	DSMZ (Germany)
S. cellulosum	DSM 53796 (So ce 56)	Prof. Rolf Müller
S. aurantiaca	DW4/3-1	(Cayuela, et al. 2003)
M. xanthus derivatives		
MR1778	Δ*carA* Δ*carH* Δ*carS*	(Ortiz-Guerrero, et al. 2011)
MR2648	Δ*carA* Δ*carH* Δ*carS* Δ*pduO*	This work
MR2649	Δ*carA* Δ*carH* Δ*carS* Δ*pduO* P_{crt}::*lacZ* (*carB*::Tn*5*-*lac*-132). Tet^R	This work
MR2915	pMR4316 (P_{van}::*carH*$_{Mx}$) in MR2649. Km^R Tet^R	This work
MR2916	pMR4426 (P_{van}::*carH*$_{Cfu}$) in MR2649. Km^R Tet^R	This work
MR2917	pMR4432 (P_{van}::*carH*$_{Ho}$) in MR2649. Km^R Tet^R	This work
MR2919	pMR4434 (P_{van}::*carH*$_{Sc}$) in MR2649. Km^R Tet^R	This work
MR3202	pMR4376 (P_{van}::*carA*$_{Mx}$) in MR2649. Km^R Tet^R	This work
MR3403	pMR4916 (P_{van}::*carH*$_{Cfe}$) in MR2649. Km^R Tet^R	This work
MR3465	pMR5011 (P_{van}::*carH*$_{Sa}$) in MR2649. Km^R Tet^R	This work
**Plasmids**		
pET15b	Vector for protein overexpression. Amp^R	Novagen
pKT25	Vector for C-terminal fusion to the T25 fragment of CyaA. Km^R	(Karimova, et al. 2000)
pMR2625	pET15b construct for overexpressing His_6-$CarA_{Mx}$. Amp^R	(López-Rubio, et al. 2002)
pMR3679	Vector for vanillate-inducible (from promoter P_{van}) expression. Km^R	(Iniesta, et al. 2012)
pMR3813	pUT18C-*carH*$_{Cc}$. Amp^R	This work
pMR3814	pUT18C-*carA*$_{Cc}$. Amp^R	This work
pMR3835	pKT25-*carA*$_{Cc}$. Km^R	This work

pMR3836	pKT25-*carH*$_{Cc}$. Km^R	This work
pMR3873	pUT18C-*carA*$_{Mf}$. Amp^R	This work
pMR3874	pUT18C-*carH*$_{Mf}$. Amp^R	This work
pMR3886	pKT25-*carA*$_{Mf}$. Km^R	This work
pMR3887	pKT25-*carH*$_{Mf}$. Km^R	This work
pMR3904	pUT18C-*carH*$_{Sc}$. Amp^R	This work
pMR3905	pKT25-*carH*$_{Sc}$. Km^R	This work
pMR3988	pUT18C-*carA*$_{Mx}$. Amp^R	This work
pMR3989	pUT18C-*carH*$_{Mx}$. Amp^R	This work
pMR4004	pKT25-*carA*$_{Mx}$. Km^R	This work
pMR4005	pKT25-*carH*$_{Mx}$. Km^R	This work
pMR4066	pUT18C-*carH*$_{Ho}$. Amp^R	This work
pMR4067	pKT25-*carH*$_{Ho}$. Km^R	This work
pMR4138	pUT18C-*carH*$_{Pp}$. Amp^R	This work
pMR4139	pKT25-*carH*$_{Pp}$. Km^R	This work
pMR4145	pUT18C-*carA*$_{Sa}$. Amp^R	This work
pMR4146	pKT25-*carA*$_{Sa}$. Km^R	This work
pMR4147	pUT18C-*carH*$_{Sa}$. Amp^R	This work
pMR4148	pKT25-*carH*$_{Sa}$. Km^R	This work
pMR4249	pET15b construct for overexpressing His$_6$-CHo (CarH$_{Ho}$DBD-CarH$_{Tt}$CBD chimera). Amp^R	This work
pMR4250	pET15b construct for overexpressing His$_6$-CSc (CarH$_{Sc}$DBD-CarH$_{Tt}$CBD chimera). Amp^R	This work
pMR4263	pUT18C-*carH*$_{Cfu}$. Amp^R	This work
pMR4264	pKT25-*carH*$_{Cfu}$. Km^R	This work
pMR4316	pMR3679-derived for P$_{van}$::*carH*$_{Mx}$ expression. Km^R	This work
pMR4421	pET15b construct for overexpressing His$_6$-CarH$_{Cfu}$. Amp^R	This work
pMR4426	pMR3679-derived for P$_{van}$::*carH*$_{Cfu}$ expression. Km^R	This work
pMR4432	pMR3679-derived for P$_{van}$::*carH*$_{Ho}$ expression. Km^R	This work
pMR4434	pMR3679-derived for P$_{van}$::*carH*$_{Sc}$ expression. Km^R	This work
pMR4605	pUT18C-*carH*$_{Av}$. Amp^R	This work
pMR4606	pKT25-*carH*$_{Av}$. Km^R	This work
pMR4607	pUT18C-*carA*$_{Av}$. Amp^R	This work
pMR4608	pKT25-*carA*$_{Av}$. Km^R	This work
pMR4808	pET15b construct for overexpressing His$_6$-CarS$_{Cfu}$. Amp^R	This work
pMR4904	pUT18C-*carH*$_{Cfe}$. Amp^R	This work
pMR4905	pKT25-*carH*$_{Cfe}$. Km^R	This work
pMR4906	pUT18C-*carH*$_{Mb}$. Amp^R	This work
pMR4907	pKT25-*carH*$_{Mb}$. Km^R	This work
pMR4912	pET15b construct for overexpressing His$_6$-CarH$_{Cfe}$. Amp^R	This work
pMR4916	pMR3679-derived for P$_{van}$::*carH*$_{Cfe}$ expression. Km^R	This work
pMR5011	pMR3679-derived for P$_{van}$::*carH*$_{Sa}$ expression. Km^R	This work

pUT18C	Vector for C-terminal fusions to the T18 fragment of CyaA. Amp^R	(Karimova, et al. 2000)

* Km^R: kanamycin resistance; Tet^R: tetracycline resistance; Amp^R: ampicillin resistance. DSMZ (German Collection of Microorganisms and Cell Cultures).

Table S4. Primers used for qRT-PCR

Myxobacteria	Gene	Primer Name	Sequence
	crtE	CfCrtE.Fw	TCGCCACCCTGCTTCGT
C. fuscus	*crtE*	CfCrtE.Rv	GGCCATCAACGGGAAGGT
	rpoD	CfSigA.Fw	GCAACATCGGCCTGATGAA
	rpoD	CfSigA.Rv	TGGCGTAGGTCGAGAACTTGT
	crtE	CfeCrtE.Fw	TGCAGGCGAGCCAGTACTG
C. ferrugineus	*crtE*	CfeCrtE.Rv	CCCCTCGCGCACCAT
	rpoD	CfeSigA.Fw	GCAACATCGGCCTGATGAA
	rpoD	CfeSigA.Rv	TGGCGTAGGTCGAGAACTTGT
	carH	ScCarH.Fw	GCCTGTACAGCGACGACGAT
	carH	ScCarH.Rv	CACGTGGTCGCGCATCT
	crtI	ScCrtI.Fw	GGAACCCGTTCTCGACGA
	crtI	ScCrtI.Rv	CCAGTTGCGCTCGAGGAA
S. cellulosum	*crtD*	ScCrtD.Fw	GATAAACACACCCCCTCATCGA
	crtD	ScCrtD.Rv	CGAACCGCTGCAGTTGCT
	crtE	ScCrtE.Fw	AGGCGTACCAGGTCGCG
	crtE	ScCrtE.Rv	CATGTCCTCCGGCCTCC
	rpoD	ScSigA.Fw	GGAACATCGGCCTCATGAAG
	rpoD	ScSigA.Rv	CGCGTACGTCGAGAACTTGTAG

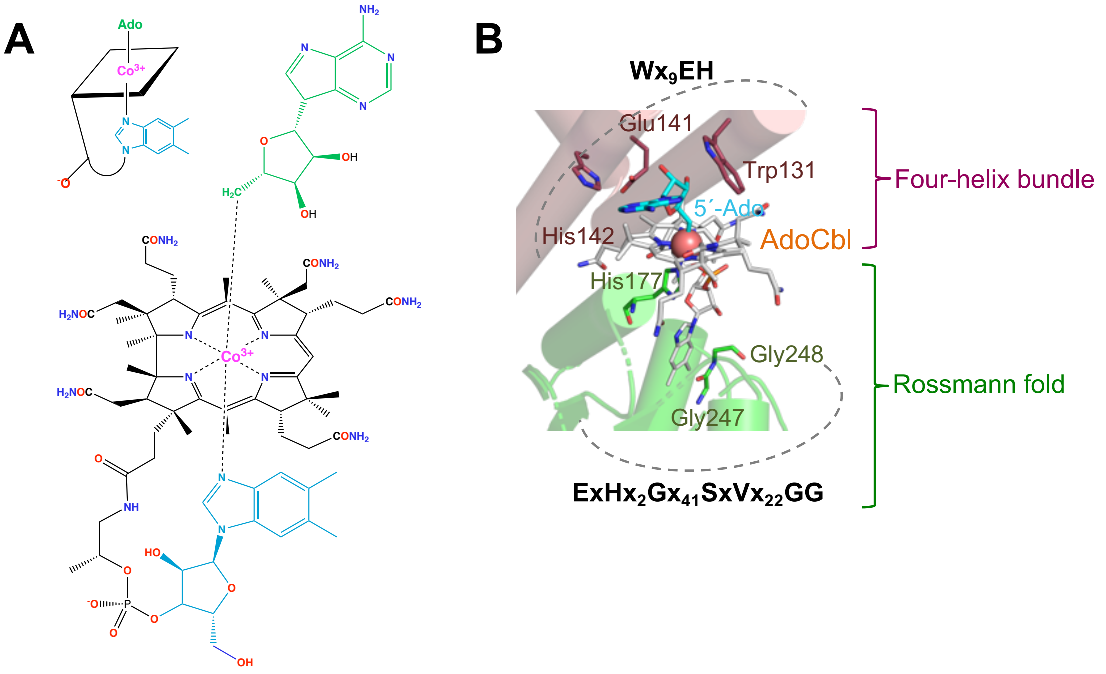

Figure S1. AdoCbl, its binding site in CarH$_{Tt}$ and distribution of CarH/A homologs among bacteria. (**A**) Chemical structure of AdoCbl in the base-on conformation, with the central cobalt atom in the Co^{+3}/Co(III) state, the lower axial ligand dimethylbenzimidazole (cyan), and the upper axial ligand 5´-deoxyadenosine (Ado) group (green), with a simpler schematic representation shown on the top left. (**B**) The CarH AdoCbl binding site in the CarH$_{Tt}$ crystal structure (PDB code: 5C8D) highlighting the Wx_9EH motif (in the four-helix bundle subdomain) that contacts the Ado group, and the His (and Gly-Gly of the ExHx_2Gx_{41}SxVx_{22}GG motif in the Rossmann fold subdomain) that serves as the lower axial ligand in the base-off/His-on B_{12}-binding mode.

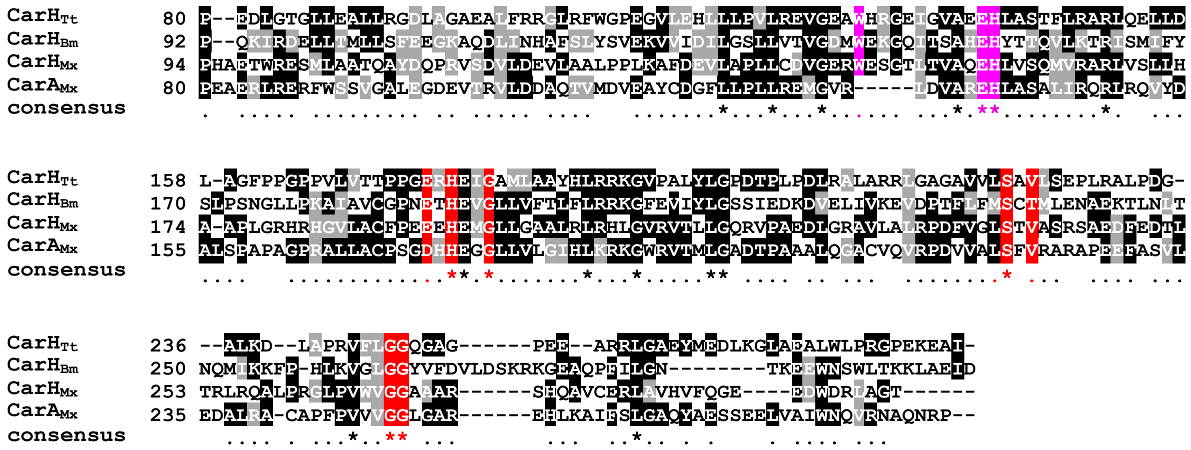

Figure S2. Sequence alignment of the B_{12}-binding domains of $CarH_{Tt}$, $CarH_{Bm}$, $CarH_{Mx}$ and $CarA_{Mx}$. Identical residues are shaded black and similar residues gray. The asterisk in the consensus line below indicates residues conserved in all four homologs. In the CarH homologs, the Ado group of bound AdoCbl is capped by the Wx_9EH motif (in magenta), which in $CarA_{Mx}$ lacks the conserved Trp and four additional less conserved residues. Conserved residues of the base-off/His-on B_{12}-binding motif are in red. NCBI accession codes are: WP_011174503 ($CarH_{Tt}$), AJI20494 ($CarH_{Bm}$), CAA79965 ($CarH_{Mx}$), CAA79964 ($CarA_{Mx}$).

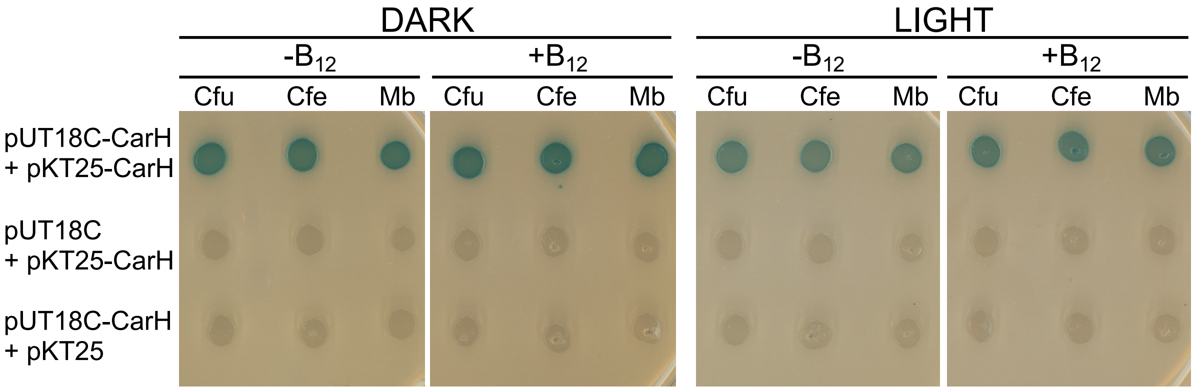

Figure S3. BACTH analysis of $CarH_{Cfu}$, $CarH_{Cfe}$ and $CarA_{Mb}$ self-interaction. Cells expressing T25 and T18 fusions of CarH or, as negative controls, only one of the two fusion proteins, were spotted on X-Gal-LB plates with or without vitamin B_{12} and incubated for 48 h in the dark or exposed to light.

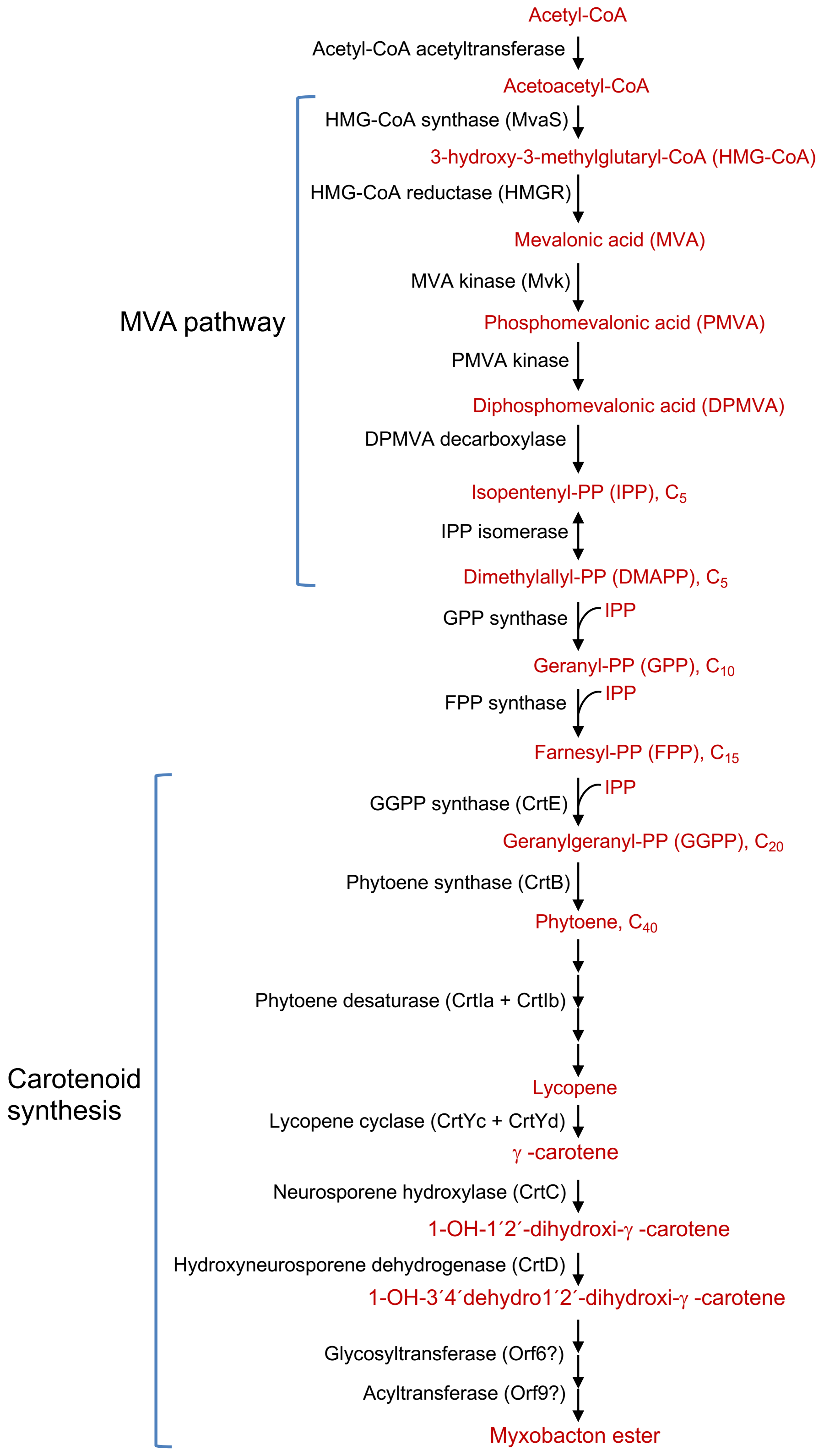

Figure S4. The MVA and carotenoid biosynthesis pathways in *M. xanthus*. The MVA pathway from acetyl-CoA to IPP/DMAPP, the essential isoprenoid precursors that feed into pathways for the synthesis of carotenoids (shown in the figure), steroids and hopanoids. The *M. xanthus* carotenoid biosynthesis pathway showing the enzymes involved and their products. PP: pyrophosphate.

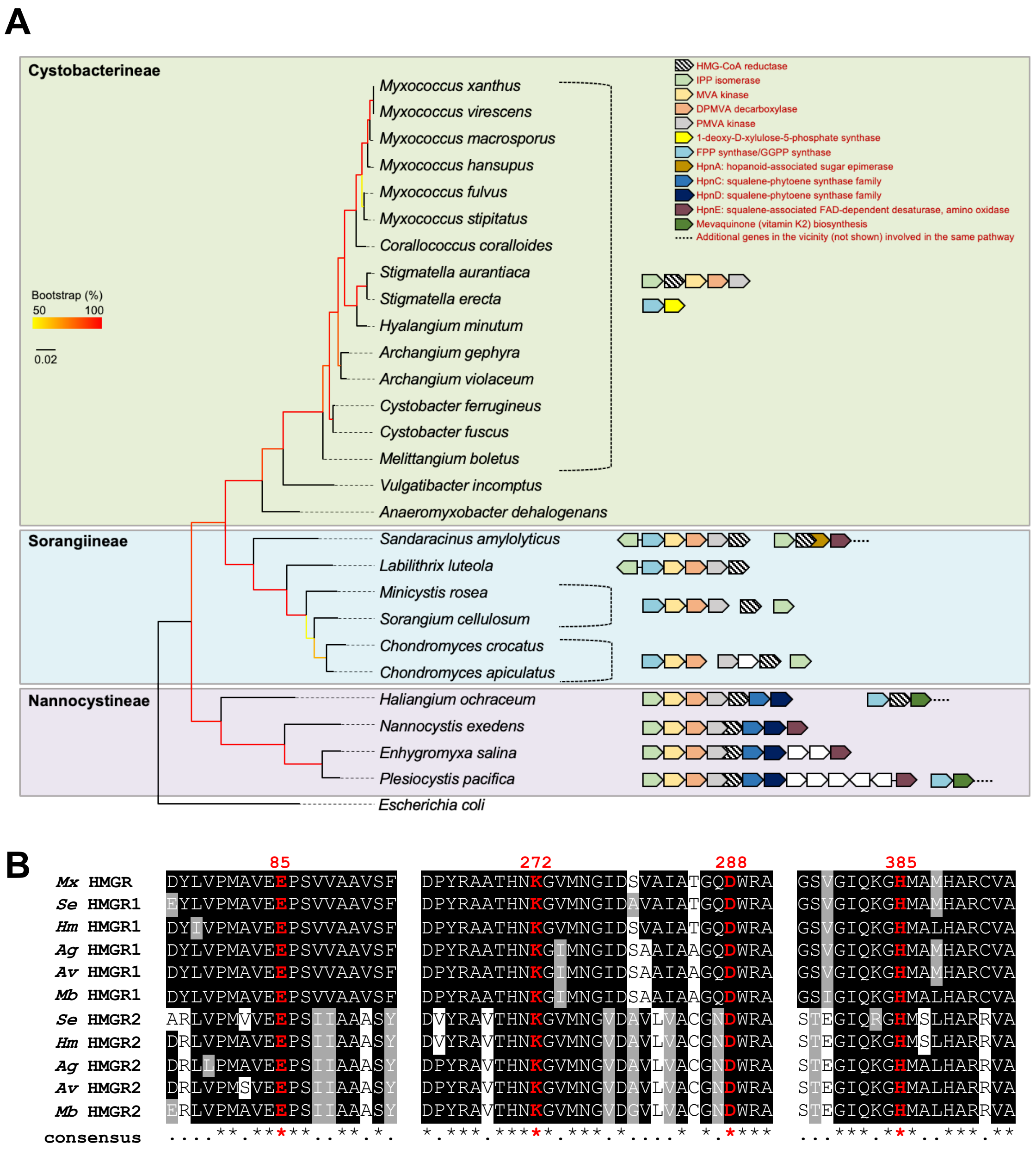

Figure S5. **Genomic arrangement of MVA pathway genes in myxobacteria**. (**A**) 16S rRNA-based phylogenetic tree highlighting the three myxobacterial suborders and species, and genome contexts of MVA pathway genes. The MVA pathway genes exist in all the myxobacterial genomes examined except for *A. dehalogenans* and *V. incomptus*, both of which have the MEP (methylerythritol 4-phosphate) pathway. Genes encoding assigned enzymes are indicated, those unfilled being of unknown function or unrelated to isoprenoid biosynthesis. (**B**) Sequence alignment of the single HMGR in *M. xanthus* and the two that occur in *S. erecta*, *H. minutum*, *A. gephyra*, *A. violaceum* and *M. boletus* showing the regions with conserved catalytic residues (in red and numbered as in *M. xanthus*). In the five myxobacteria with two HMGR paralogs, the gene for the primary HMGR (HMGR/HMGR1) is found clustered with other key genes for MVA pathway enzymes, and the gene for the second HMGR (HMGR2) is in the cluster of carotenoid synthesis genes.

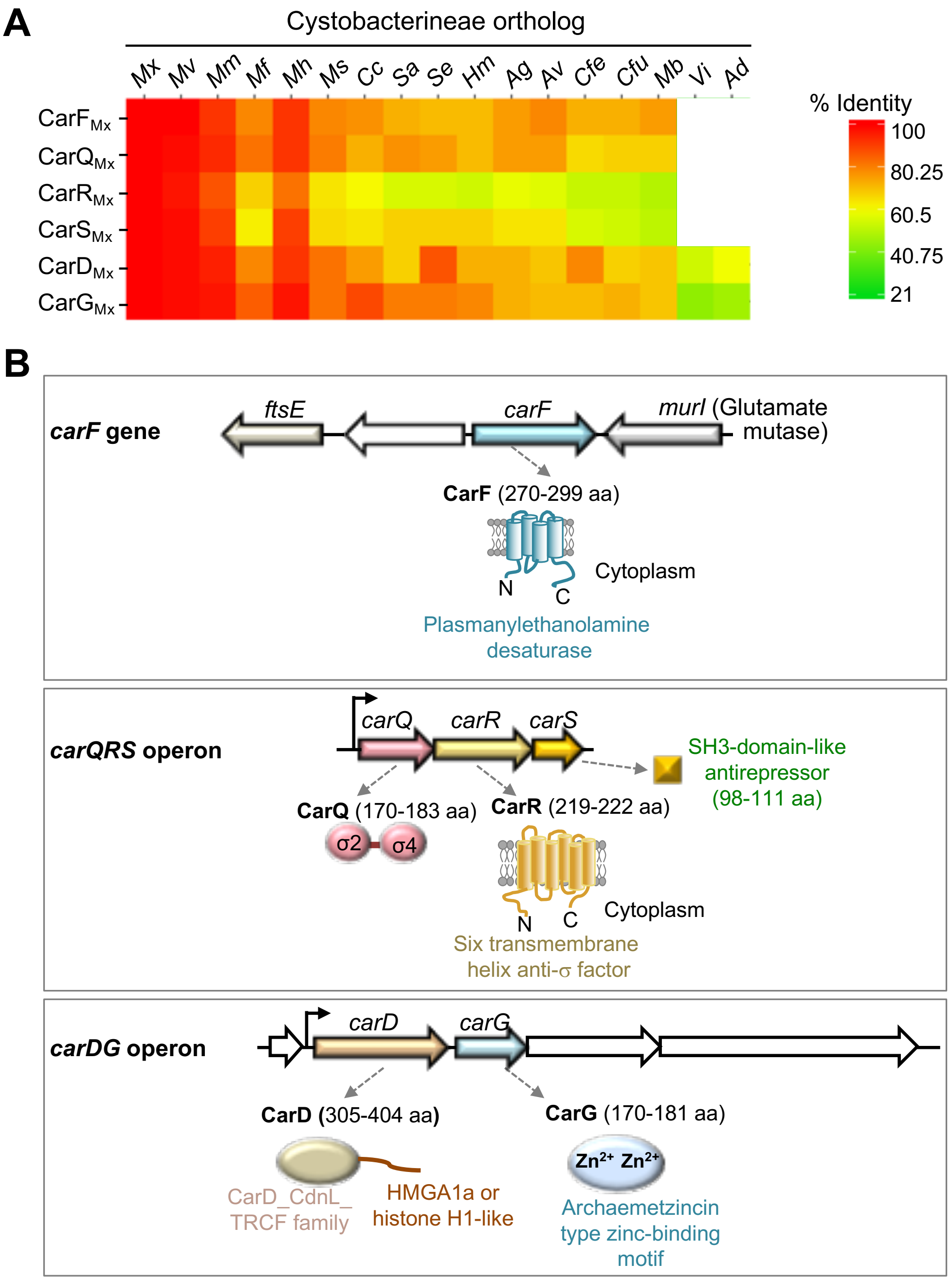

Figure S6. **Comparisons of *M. xanthus* CarF, CarQ, CarR, CarS, CarD and CarG with orthologs in Cystobacterineae, and their genome contexts.** (**A**) Heat map of the sequence percent identities of *M. xanthus* CarF, CarQ, CarR, CarS, CarD and CarG and the corresponding myxobacterial orthologs, which are found in suborder Cystobacterineae. Species are abbreviated as in Table S1. (**B**) Genomic context in Cystobacterineae of genes encoding: CarF (top); CarQ, CarR and CarS (middle); CarD and CarG (bottom) in *M. xanthus*. Genes are colored if they are known to participate in the CarA pathway, grey if annotated but without any known role in the pathway, and unfilled for hypothetical proteins of unknown function. Proteins, their size range (number of amino acids) and domain organization are shown schematically: CarQ with ECF-σ subdomains σ2 and σ4; CarR and CarF with its six and four transmembrane-helix topologies, respectively; the two CarD domains and CarG with its two bound zinc atoms.

Cystobacterineae

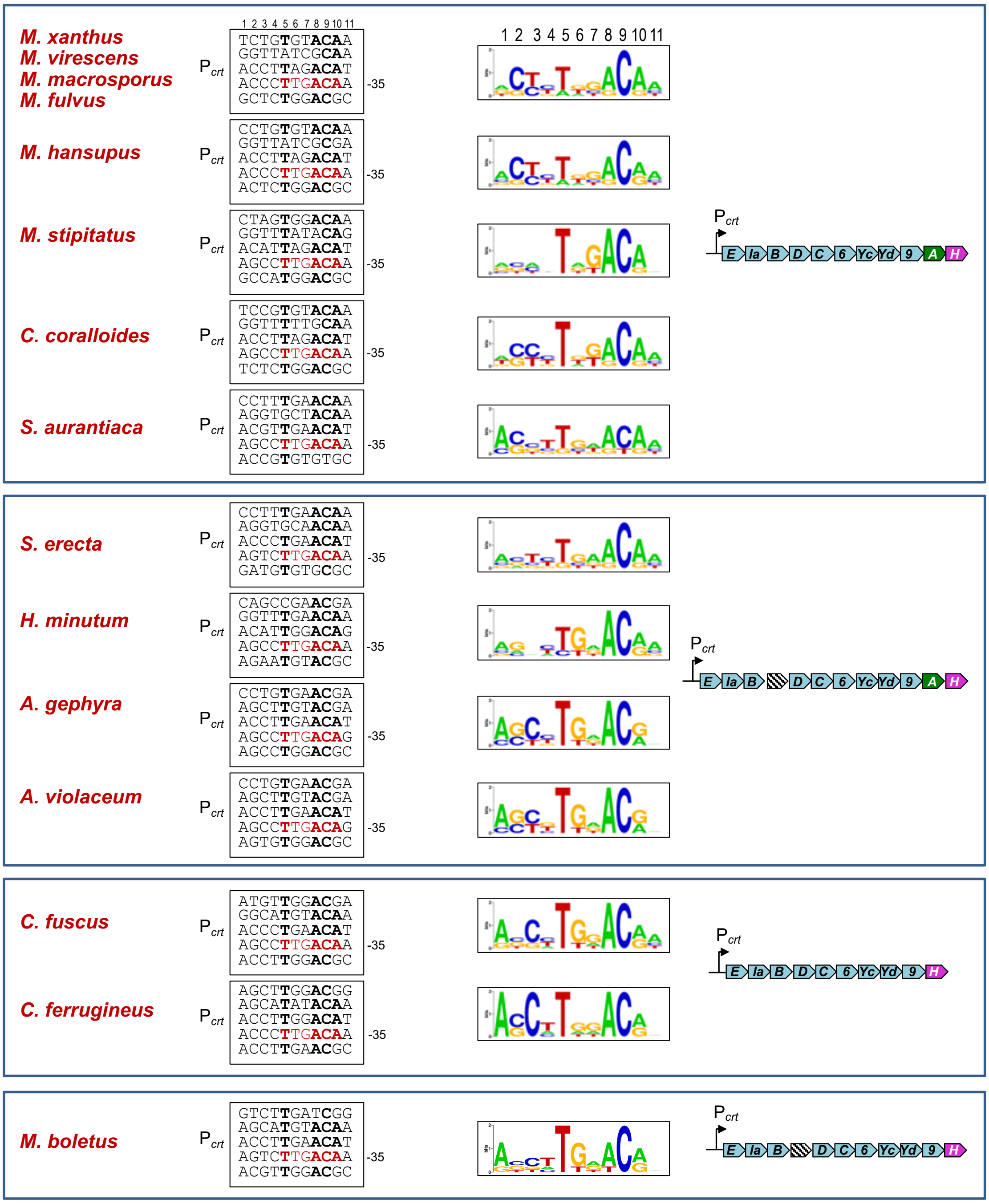

Figure S7. **Comparison of the P*crt* region in Cystobacterineae.** Sequence logos based on the five 11-bp tandem direct repeats at P*crt* in Cystobacterineae shown on the left. The five repeats are shown with the conserved T and ACA of the T*nn*ACA motif in boldface, and the -35 TTGACA element (identical in all the species shown) in red. See figure 4 legend for genes and their products.

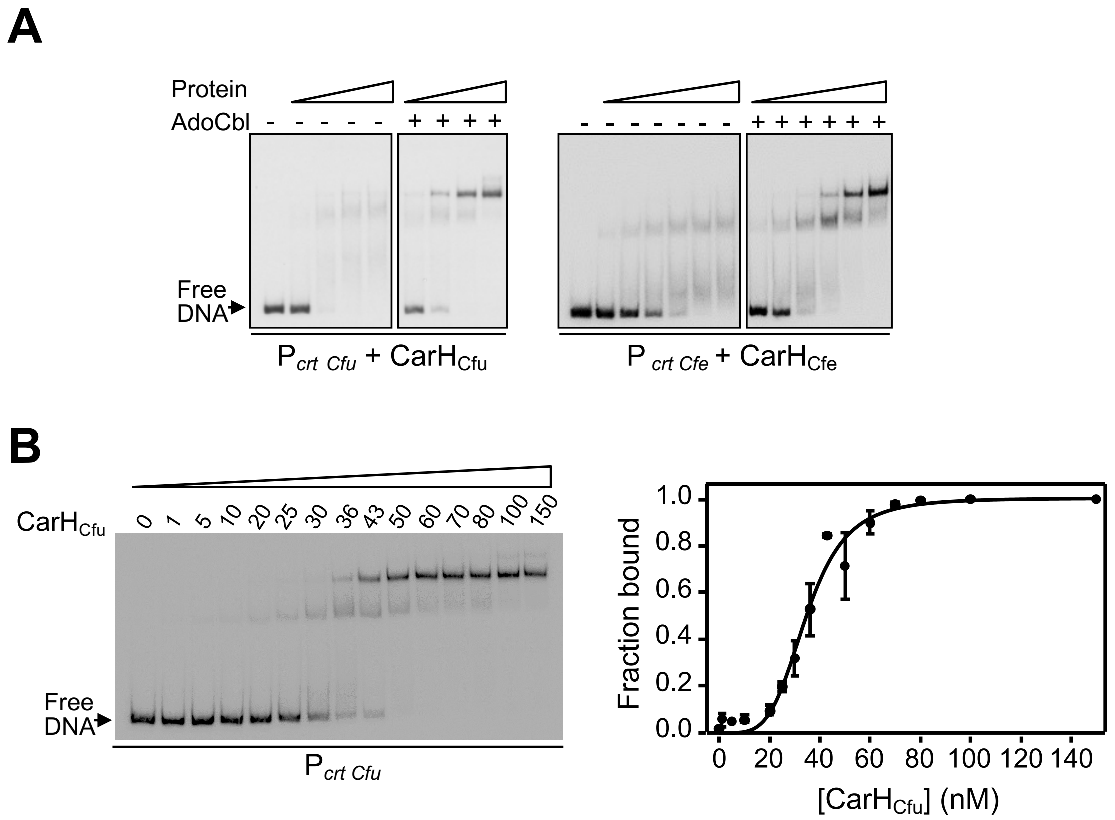

Figure S8. **DNA binding of *Cystobacter* CarH**. **(A)** Representative EMSA for the binding of $CarH_{Cfu}$ (25, 50, 100, 200 nM) to the 170-bp $P_{crt\ Cfu}$ probe and of $CarH_{Cfe}$ (3, 6, 12, 25, 50, 100 nM) to the 170-bp $P_{crt\ Cfe}$ probe in the dark with or without AdoCbl. **(B)** Representative EMSA titration of the binding of $CarH_{Cfu}$ to $P_{crt\ Cfu}$ in the dark and presence of AdoCbl (left) and fit of the data from three independent titrations to the Hill equation (right). The fit yields K_D ($CarH_{Cfu}$ concentration at half-maximal binding) of 34.7±1.2 nM and a Hill coefficient of 4.3±0.6.

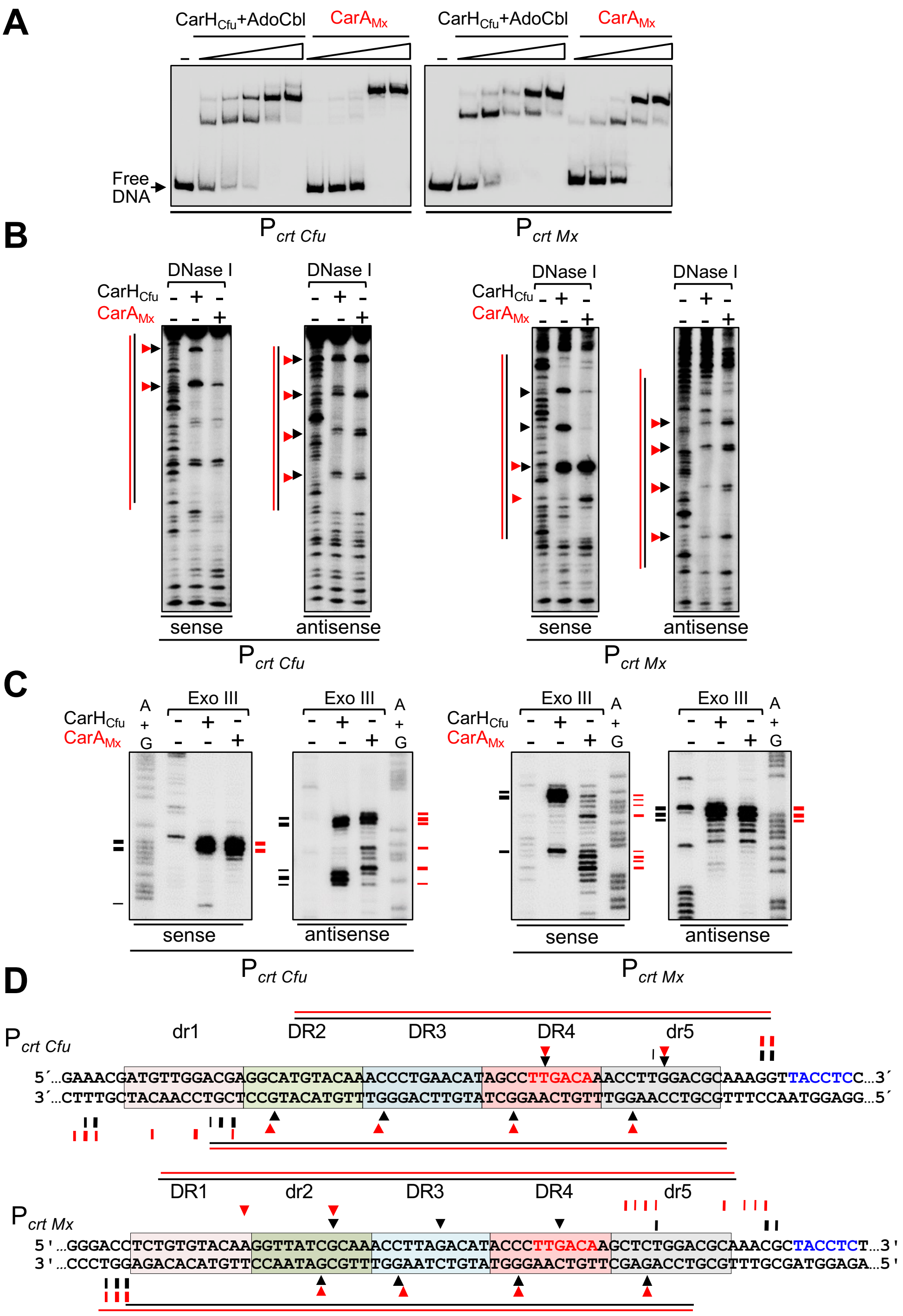

Figure S9. **Comparison of the binding of CarH$_{Cfu}$ and CarA$_{Mx}$ to P$_{crt\ Cfu}$ and P$_{crt\ Mx}$. (A)** Representative EMSA for binding in the dark of CarH$_{Cfu}$ (in the presence of AdoCbl) and CarA$_{Mx}$ to probes P$_{crt\ Cfu}$ and P$_{crt\ Mx}$. (**B**) DNase I footprints on the sense and antisense strands for the binding of CarH$_{Cfu}$ (in the presence of AdoCbl) and CarA$_{Mx}$ in the dark to probes P$_{crt\ Cfu}$ and P$_{crt\ Mx}$. Lines indicate DNase I footprints and arrowheads point to DNase I hypersensitive sites. (**C**) Representative Exo III footprints on the sense and antisense strands for the binding of CarH$_{Cfu}$ (in the presence of AdoCbl) and CarA$_{Mx}$ in the dark to probes P$_{crt\ Cfu}$ and P$_{crt\ Mx}$. Positions of Exo III arrest align with the horizontal lines on the side (thicker lines for stronger arrest). (**D**) Summary of footprint data on each strand (sense, top; antisense, bottom) for the two probes. Horizontal lines span the DNase I footprints, vertical lines point to positions of Exo III arrest (thicker for stronger arrest), and arrowheads to DNase I

hypersensitive sites. Each 11-bp repeat is boxed, shaded and labeled (uppercase: conforms to 5′-*nnnn*T*nn*ACA*n*-3′ consensus; lowercase: divergent) with numbering (1 to 5) relative to the sense strand. The -35 and -10 promoter elements are lettered in red and blue, respectively. All footprints (indicated in black for $CarH_{Cfu}$, in red for $CarA_{Mx}$) were mapped using the corresponding A+G sequence ladder.

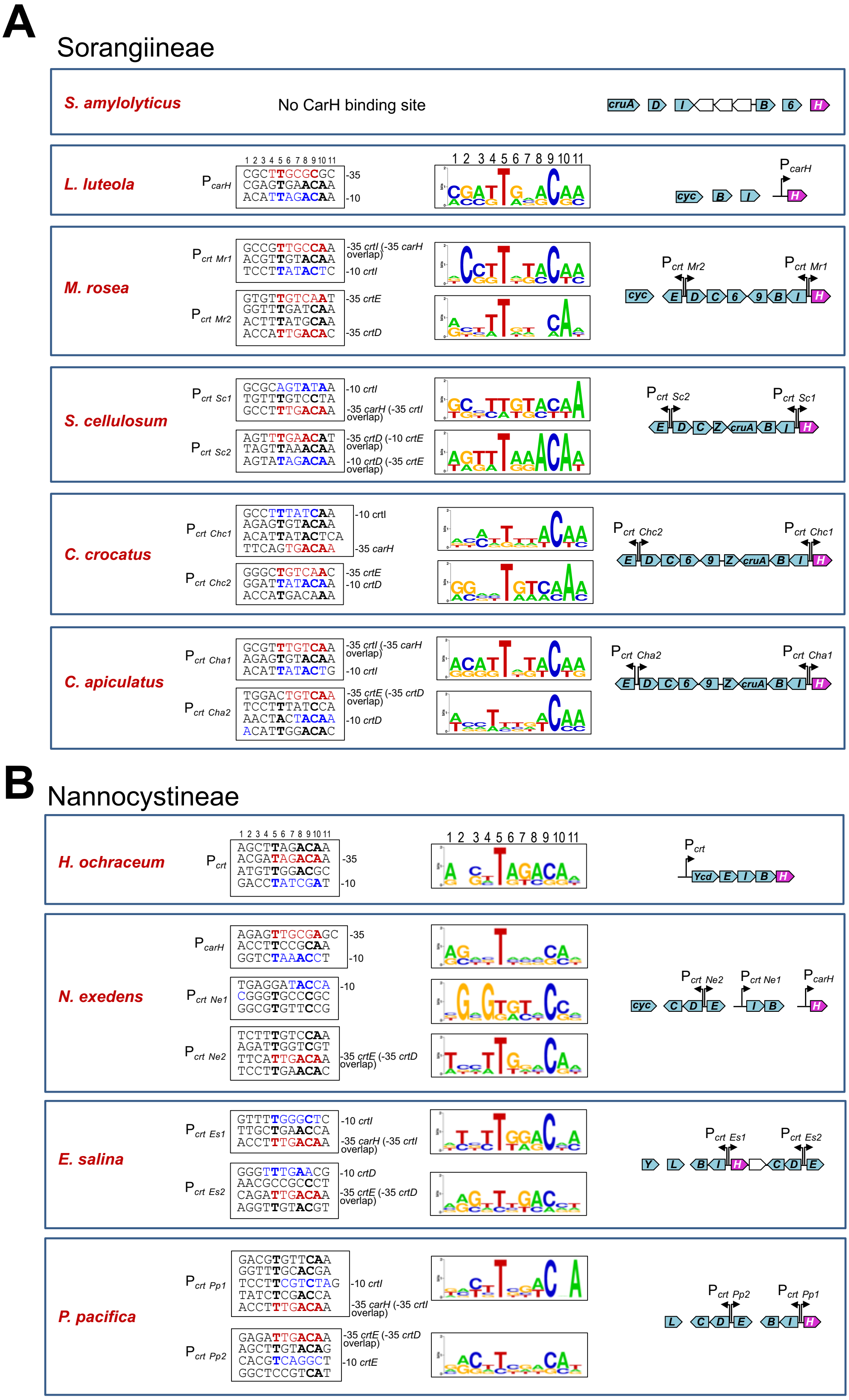

Figure S10. **Possible CarH binding sites at P$_{crt}$ promoter regions in Sorangiineae and Nannocystineae.** Sequence logos based on the 11-bp tandem direct repeats found at the indicated P$_{crt}$ promoters in Sorangiineae (**A**) and Nannocystineae (**B**) are shown. Conserved bases of the T*nn*ACA motif are in boldface, and the tentative -35 and -10 promoter elements are in red and blue, respectively. See figure 4 legend for genes and their products.

Supporting Information References

Cayuela ML, Elías-Arnanz M, Peñalver-Mellado M, Padmanabhan S, Murillo FJ. 2003. The *Stigmatella aurantiaca* homolog of *Myxococcus xanthus* high-mobility-group A-type transcription factor CarD: insights into the functional modules of CarD and their distribution in bacteria. *J Bacteriol.* 185:3527-3537.

Hanahan D. 1983. Studies on transformation of *Escherichia coli* with plasmids. *J Mol Biol.* 166:557-580.

Iniesta AA, García-Heras F, Abellón-Ruiz J, Gallego-García A, Elías-Arnanz M. 2012. Two systems for conditional gene expression in *Myxococcus xanthus* inducible by isopropyl-β-D-thiogalactopyranoside or vanillate. *J Bacteriol.* 194:5875-5885.

Karimova G, Ullmann A, Ladant D. 2000. A bacterial two-hybrid system that exploits a cAMP signaling cascade in *Escherichia coli*. *Methods Enzymol.* 328:59-73.

López-Rubio JJ, Elías-Arnanz M, Padmanabhan S, Murillo FJ. 2002. A repressor-antirepressor pair links two loci controlling light-induced carotenogenesis in *Myxococcus xanthus*. *J Biol Chem.* 277:7262-7270.

Ortiz-Guerrero JM, Polanco MC, Murillo FJ, Padmanabhan S, Elías-Arnanz M. 2011. Light-dependent gene regulation by a coenzyme B_{12}-based photoreceptor. *Proc Natl Acad Sci USA.* 108:7565-7570.

Ruiz-Vázquez R, Murillo FJ. 1984. Abnormal motility and fruiting behavior of *Myxococcus xanthus* bacteriophage-resistant strains induced by a clear-plaque mutant of bacteriophage Mx8. *J Bacteriol.* 160:818-821.

Shelton AN, Seth EC, Mok KC, Han AW, Jackson SN, Haft DR, Taga ME. 2019. Uneven distribution of cobamide biosynthesis and dependence in bacteria predicted by comparative genomics. *ISME J.* 13:789-804.

Yanisch-Perron C, Vieira J, Messing J. 1985. Improved M13 phage cloning vectors and host strains: nucleotide sequences of the M13mp18 and pUC19 vectors. *Gene.* 33:103-119.
